# Influence of race, ethnicity, and sex on the performance of epigenetic predictors of phenotypic traits

**DOI:** 10.1186/s13148-025-01864-6

**Published:** 2025-04-09

**Authors:** Dennis Khodasevich, Nicole Gladish, Saher Daredia, Anne K. Bozack, Hanyang Shen, Jamaji C. Nwanaji-Enwerem, Belinda L. Needham, David H. Rehkopf, Andres Cardenas

**Affiliations:** 1 Department of Epidemiology and Population Health, Stanford University, Palo Alto, CA 94305 USA; 2 Division of Epidemiology, Berkeley Public Health, University of California, Berkeley, Berkeley, CA USA; 3 Department of Emergency Medicine, Center for Health Justice, and Center of Excellence in Environmental Toxicology, Perelman School of Medicine, University of Pennsylvania, Philadelphia, PA USA; 4 Department of Epidemiology, University of Michigan, Ann Arbor, MI USA; 5 Department of Health Policy, Stanford University, Palo Alto, CA USA; 6 Department of Medicine (Primary Care and Population Health), Stanford University, Palo Alto, CA USA; 7 Department of Pediatrics, Stanford University, Palo Alto, CA USA; 8 Department of Sociology, Stanford University, Palo Alto, CA USA

**Keywords:** Epigenetics, Aging, Machine learning, Disparities

## Abstract

**Background:**

DNA methylation-based predictors of phenotypic traits including leukocyte proportions, smoking activity, biological aging, and circulating levels of plasma proteins are widely used as biomarkers in public health research. However, limited racial and ethnic diversity of research participants is an ongoing issue for epigenetics research, and the potential downstream impacts of limited diversity in training samples on the performance of epigenetic predictors remains poorly understood. We examined the performance of epigenetic predictors of chronological age (also known as epigenetic clocks), telomere length, cell proportions, and plasma proteins within a diverse sample of adult NHANES participants during the 1999–2000 and 2001–2002 survey cycles, both overall and stratified by self-reported race/ethnicity and sex. We utilized correlation coefficients and median absolute errors (MAE) to judge predictor performance, and bootstrapping and multivariate regression to assess the significance of differences between groups.

**Results:**

All epigenetic predictors were significantly associated with their corresponding phenotypic traits in the overall population, with particularly high correlations for the epigenetic clocks and cell proportion estimates. Several significant differences in performance were observed between racial/ethnic groups, particularly for the plasma protein predictors, with a reoccurring trend of lower correlation in Mexican American and non-Hispanic Black participants compared to non-Hispanic White participants. Sex-differences in performance for several predictors were also identified but were not as pronounced. Multivariate regression models indicated that disparities in epigenetic predictor performance persisted after accounting for overall differences in epigenetic predictions related to race/ethnicity and sex, as well as further adjustment for estimated cell proportions and SES variables.

**Conclusions:**

We found evidence for substantial disparities in epigenetic predictor performance, with each predictor exhibiting at least one significant difference in correlation or MAE related to race, ethnicity, or sex.

**Supplementary Information:**

The online version contains supplementary material available at 10.1186/s13148-025-01864-6.

## Background

Epigenetics is the study of modifications that influence gene expression without changes to the underlying DNA sequence and include DNA methylation (DNAm) and histone modifications. DNAm is often studied in the context of the regulation of gene expression, but DNAm data can additionally be harnessed to generate predictions of a wide variety of phenotypic traits including leukocyte proportions, smoking activity, chronological age, and circulating levels of plasma proteins [1–5]. These epigenetic predictors have proven to be invaluable biomarkers in a range of contexts including studying the determinants of biological aging, controlling for cell-type heterogeneity in epigenetic research, and even for use in place of laboratory-derived measures of plasma protein markers [6–8]. In particular, epigenetic clocks, epigenetic predictors of chronological and biological aging, have revealed substantial influence of social and environmental factors in shaping biological aging in human populations [6]. Following well-documented systemic disparities in social and environmental exposures, several studies have reported differential epigenetic aging according to race and ethnicity [9, 10]. However, limited research has sought to directly evaluate the examine the performance of epigenetic predictors in relation to key demographic variables, with just one previous analysis in the Environmental influences on Child Health Outcomes (ECHO) consortium found variable performance of several pediatric epigenetic clocks between Black and White children [11].

Participants of European ancestry are historically overrepresented in epigenetics research, and this issue extends to the diversity of populations represented in the training samples of epigenetic predictors [12]. Assembly of training samples used to develop epigenetic predictors are often based on convenience, utilizing any available datasets containing both DNAm data and phenotypic data of interest, rather than explicitly seeking to compile diverse and representative samples to ensure external validity. Diversity of training samples and reporting of key demographic variables including race/ethnicity and country of origin remain key issues in the development of several commonly used epigenetic clocks, which has been summarized in an extensive review from Watkins and colleagues [13]. This issue of limited diversity in training samples extends well beyond epigenetic predictors, with similar issues raised in the development of polygenic risk scores and models based on electronic health records, indicating that limited training sample diversity is a pervasive and salient issue across multiple health-related disciplines [14–16].

Although epigenetic predictors are widely utilized in a variety of research contexts, limited research has explicitly set out to evaluate their performance in relation to relevant demographic variables. We sought to systematically examine the performance of epigenetic predictors of cell proportion estimates, plasma protein levels, telomere length, and several predictors of chronological age in relation to self-reported race/ethnicity and sex within a diverse sample of older adult National Health and Nutrition Survey (NHANES) participants during the 1999–2000 and 2001–2002 survey cycles. NHANES is a program designed to assess the health status of a nationally representative sample of adults and children in the United States (US) and features a cross-sectional sample with high-quality laboratory measures and DNAm data in a large number of US participants, providing a unique opportunity to evaluate the performance of several categories of epigenetic predictors.

## Methods

### Study population

Our study harnessed data from the 1999–2000 and 2001–2002 cycles of NHANES, a biannual program which collects data from a representative sample of the non-institutionalized US population organized by the National Center for Health Statistics (NCHS) [17]. This subsample included 2,532 adult participants aged ≥ 50 years surveyed in 1999–2000 or 2001–2002 that had blood samples available for DNA methylation (DNAm) analysis. Self-reported race and ethnicity was encoded in 5 categories: Mexican American, Other Hispanic, Non-Hispanic (NH) White, NH Black, and Other Race—Including Multi-Racial. In the 1999–2000 and 2001–2002 NHANES survey cycles, participants ≥ 85 years of age were top-coded as 85 years of age. To prevent incorrect chronological age estimates from influencing results, all participants labeled as 85 years of age (*N* = 126) were removed for analyses related to assessing the fit of epigenetic clocks. All NHANES participants provided written informed consent, and study protocols were approved by the NCHS Research Ethics Review Board.

### DNA methylation

DNA was extracted from whole blood from a selection of NHANES adult participants aged ≥ 50 years surveyed in 1999–2000 or 2001–2002, and DNAm was measured with the Illumina EPIC BeadChip array [18]. Detailed methodology and complete code used for DNAm data processing and quality control are provided in the NHANES DNA methylation array and epigenetic biomarkers data documentation [18]. DNAm was used to generate predictions of chronological age, telomere length, circulating plasma protein levels, and blood cell proportions. Several epigenetic age predictions were calculated including Horvath’s panTissue, Hannum, Skin&Blood, Lin, Weidner, Vidal-Bralo, and Zhang clocks, as well as the DNAmTL telomere length estimator [3, 5, 19–26]. We focus analysis on epigenetic predictors of chronological age, excluding PhenoAge, GrimAge, GrimAge2, DunedinPoAm, and the Yang cell division estimator because each of these predictors were trained to predict specific aspects of the biological aging process that were not directly available among the NHANES datasets. However, we further examined the prediction accuracy of the component GrimAge and GrimAge2 plasma protein predictors. GrimAge is a multistage epigenetic clock composed of seven predictors of plasma protein levels, including Beta-2 microglobulin (B2M) and Cystatin C, and smoking pack years [5]. Recently, an updated version of GrimAge was developed, referred to as GrimAge2, incorporating the original predictors in GrimAge and two novel DNAm-based predictors of C-reactive protein (CRP) and Hemoglobin A1c (HbA1c) [26]. Because GrimAge2 components were trained to predict measures on the log scale, GrimAge2 CRP and HbA1c predictions were exponentiated prior to analysis. Furthermore, DNAm was used to predict proportions of six cell types (neutrophils, monocytes, B-lymphocytes, natural killer cells, CD4+ , and CD8 + T-cells) [1, 27, 28]. To limit the influence of potentially mislabeled samples, we excluded *N* = 60 participants whose DNAm-derived sex prediction did not match their reported sex.

### Laboratory measurements

B2M, Cystatin C, and CRP were measured in serum, and HbA1c % was measured in blood in the NHANES study population during the 1999–2000 and 2001–2002 survey cycles [29–33]. For ease of interpretation, all laboratory-derived and DNAm-derived plasma protein concentrations were converted to mg/L, except HbA1c which is expressed as a percentage. Telomere length, expressed as the mean T/S ratio (measured telomere length relative to standard reference DNA), was measured in blood in the NHANES study population and converted to kilobase pairs using the provided formula: (3274 + 2413 × (T/S))/1000 [34]. Complete blood cell counts were generated using the Beckman Coulter method of counting and sizing for all participants with available blood samples [35, 36]. We compared laboratory-derived monocyte proportions to DNAm-derived monocyte proportions, laboratory-derived segmented neutrophil proportions to DNAm-derived neutrophils proportions, and, because laboratory-derived and DNAm-derived cell proportions differed in granularity of cell type estimates, we compared laboratory-derived lymphocyte proportions to the sum of DNAm-derived proportions of B-lymphocytes, CD4+ , and CD8 + T-cells. A majority of participants had each laboratory measure available, but a small portion of participants with missing values for specific laboratory measures were excluded from corresponding analyses, totaling up to *N* = 28 missing Cystatin C, *N* = 31 missing B2M, *N* = 1 missing HbA1c, *N* = 2 missing telomere length, and *N* = 13 missing cell proportion measures. Furthermore, to prevent undue influence from extreme outliers, we further excluded observations with laboratory-derived variable measures ≥ 3 standard deviations above or below the mean on the log scale, leading to the removal of *N* = 39 Cystatin C measures, *N* = 43 B2M measures, *N* = 10 CRP measures, *N* = 63 HbA1c measures, *N* = 27 neutrophil measures, *N* = 21 lymphocyte measures, *N* = 22 monocyte measures, and *N* = 13 telomere length measures.

### Statistical analysis

We evaluated fit between laboratory-derived and DNAm-derived biomarker measures using Pearson correlation coefficients and median absolute errors (MAE), both in the overall population and stratified by race/ethnicity category or sex. We focus most interpretation on Pearson correlation coefficients because correlation is often used as a primary metric to evaluate the accuracy of epigenetic predictors in novel study populations, as well as criteria for the evaluation of epigenetic predictor performance in training/testing samples [4, 5]. We used bootstrapping to evaluate the significance of differences in correlation and MAE between laboratory-derived and DNAm-derived biomarker measures between each group, using 10,000 iterations of sampling with replacement using the R *sample* function, then extracting the median difference across all iterations as well as the 2.5th and 97.5th percentiles to generate a 95% confidence interval (CI). Due to smaller sample sizes within the Other Hispanic and Other/Multiracial categories, we focus bootstrapped race/ethnicity-stratified analyses on NH White, NH Black, and Mexican American groups. Bootstrapped differences were considered significant if the 95% CI did not include the null value of 0. Furthermore, as a sensitivity analysis to account for the potential influence of differences in sample size between groups, we performed 10,000 iterations of sampling with replacement as described above, while setting the number of sampled observations within each group equal to the number of observations in the smallest group, which was the NH Black group in the race/ethnicity analysis (maximum *N* = 533) and female participants in the sex analysis (maximum *N* = 1217).

To further examine the influence of race/ethnicity on associations between phenotypic traits and their corresponding epigenetic predictions, we utilized multivariate linear regression models with the epigenetic prediction as the outcome; the phenotypic trait, race/ethnicity, an interaction term between race/ethnicity and the phenotypic trait as the predictor variables, further adjusted for sex. Additional models were also run to examine the influence of sex, with the epigenetic prediction as the outcome; the phenotypic trait, sex, an interaction term between sex and the phenotypic trait as the predictor variables, further adjusted for race/ethnicity. NH White participants were encoded as the reference group for the race/ethnicity analysis and female participants were encoded as the reference group in the sex analysis. In these models, the phenotype model term represents the expected change in epigenetic prediction for a 1 unit change in the phenotypic trait within the reference group, where a regression coefficient of 1 would indicate a perfect linear association between the phenotypic trait and the epigenetic prediction, while the interaction term represents the additional expected change in the association between the phenotypic and the epigenetic prediction within the specified group. Additionally, to examine the potential influence of cell heterogeneity and socioeconomic variables in modifying these associations, we reran these models, excluding the cell proportion estimators, and further adjusted for participant education status (< 9th grade, 9–11th grade, high school graduate/GED, some college or AA degree, college graduate or above, or don’t know), poverty-to-income ratio, and DNAm-derived estimates of CD8T, CD4T, NK cells, B cells, monocytes, and neutrophils. Bonferroni-adjusted *p*-values < 0.05, considering the number of tests to be 150 (15 predictors × 5 race/ethnicity groups × 2 sex groups) for the minimally-adjusted analysis and 120 (12 predictors × 5 race/ethnicity groups × 2 sex groups) for the fully-adjusted analysis, were judged as significant, while results with unadjusted *p*-values < 0.05 were considered suggestive. All analyses were performed in *R* version 4.2.3.

## Results

### Study sample

The majority of the NHANES study sample identified as NH White (*N* = 1005), with large portions of the study sample identifying as Mexican American (*N* = 694) or NH Black (*N* = 533), and smaller portions of the sample identifying as Other Hispanic (*N* = 159) or Other/Multiracial (*N* = 81) (Table 1). The study sample featured a relatively even balance between male (*N* = 1255) and female participants (*N* = 1217). The overall study sample consisted of adult participants aged 50 years and older (Mean Age: 66.1 years, SD: 10.1 years). However, all participants 85 years of age and older were top-coded as 85 years of age and, to prevent undue influence from mislabeled age, these participants (*N* = 126) were excluded for assessment of the fit of epigenetic predictors of chronological age.

**Table 1:** Sample demographics of adults from NHANES 1999–2000 and 2001–2002 with available DNA methylation data

Variable	Distribution
	Count (%)
*Race/Ethnicity*	
Mexican American	694 (28.1%)
Other Hispanic	159 (6.4%)
Non-Hispanic White	1,005 (40.7%)
Non-Hispanic Black	533 (21.6%)
Other/Multiracial	81 (3.3%)
*Sex*	
Male	1255 (50.8%)
Female	1217 (49.2%)

	Mean (SD)
Age (years)	66.1 (10.1)

	Corr. (MAE)
Horvath Clock	0.80 (3.44)
Hannum Clock	0.82 (3.43)
Skin&Blood Clock	0.87 (2.71)
Lin Clock	0.76 (9.04)
Vidal-Bralo Clock	0.62 (6.09)
Weidner Clock	0.55 (11.37)
Zhang Clock	0.89 (4.97)
Telomere Length	0.38 (1.12)
Beta-2 Microglobulin (B2M)	0.46 (0.46)
C-Reactive Protein (CRP)	0.35 (1.51)
Cystatin C	0.43 (0.24)
HbA1c	0.41 (0.30)
Total Lymphocytes	0.88 (0.03)
Monocytes	0.75 (0.01)
Neutrophils	0.88 (0.02)

Counts (%) are given for demographic variables, mean (SD) is provided for chronological age, and Pearson correlation coefficients (MAE) between DNAm-derived and lab-derived measures or chronological age in the overall sample are provided for all biomarkers. Unweighted distributions are presented

We further examined potential differences in the distribution of each of the phenotypic traits and demographic variables known or suspected to influence epigenetic predictions between race/ethnicity categories in the study sample (Table S1). Age distributions were similar across all groups, with the oldest average age in the NH White group (Mean 68.1, SD: 10.9). The poverty-to-income (PIR) ratio differed widely between groups, with the highest average PIR in the NH White group (Mean: 3.16, SD: 1.57) and the lowest average PIR in the Other Hispanic group (Mean: 1.58, SD: 1.20). Educational attainment also differed widely between groups, with < 9th grade education the most common category in the Mexican American (58.2%) and Other Hispanic (39.9%) groups, 9–11th grade education being most common in the NH Black group (33.0%), High School/GED being most common in the NH White group (29.4%), and College Graduate/Above being most common in the Other/Multiracial group (25.9%). Participants most commonly reported never smoking across all groups, except within the NH White group where former smoking was most commonly reported.

The epigenetic predictors considered in our analysis differed widely in the demographics of their training samples, as well as in the direct reporting of these demographics (Table S2). The Cystatin C and B2M plasma protein predictors, components of GrimAge, were trained in a sample of 1731 Framingham Heart Study (FHS) participants, with the racial/ethnic demographics not directly reported but presumed to be entirely White given the racial and ethnic composition of the general FHS cohort. The DNAmTL telomere length estimator was trained in a sample of 2256 Women’s Health Initiative and Jackson Heart Study participants, with a majority of the sample identifying as African American (81%) and female (75%). Several of the epigenetic predictors did not directly report demographics of their training samples beyond chronological age, sex, and tissue type or were trained on collections of public datasets each with varied reporting on demographics. For example, ~ 26.3% of the training samples used to develop the Horvath clock were missing reported information on participant race or ethnicity. The cell proportion estimator training sample featured some of the most detailed demographic reporting, with 16% of the sample identifying as female; and the sample consisting of 56.8% Indo-European, 16.2% African American, 21.6% Mixed, and 5.4% East Asian participants.

### Overall assessment

**Fig. 1:**
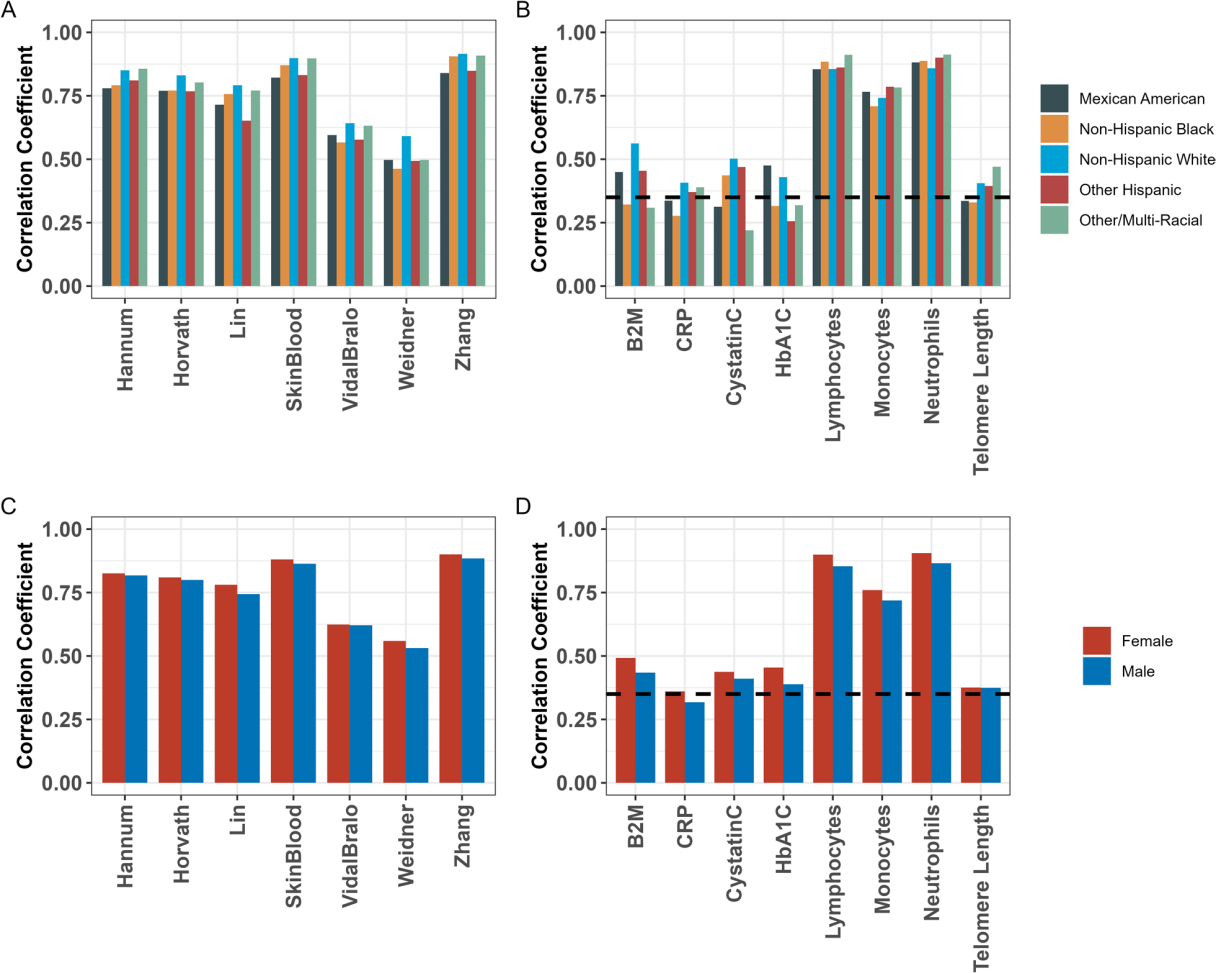
Pearson correlation coefficients for epigenetic predictors, stratified by race/ethnicity category (**A**, **B**) or stratified by sex (**C**, **D**). Dotted line in panels (**B**) and (**D**) displays the 0.35 correlation cutoff originally used to evaluate GrimAge plasma protein predictors

Most epigenetic clocks exhibited relatively high correlations with chronological age across all categories of reported race/ethnicity and sex, with the highest correlations observed with the Zhang clock among NH White participants (*r* = 0.92) and Other/Multiracial participants (*r* = 0.91), and the lowest correlation observed with the Weidner clock (*r* = 0.46) within the NH Black group (Fig. 1). The highest correlation for the telomere length estimator was seen within the Other/Multiracial group (*r* = 0.47), and the lowest correlation was seen within the NH Black group (*r* = 0.33). The highest median absolute errors (MAE) were observed with the Weidner and Lin clocks, while the lowest MAE was observed with the Skin&Blood clock (Fig. S1). Cell proportion estimates generally also performed well across all groups, with the highest correlation observed with neutrophils within the Other/Multiracial category (*r* = 0.91) and the lowest correlation with monocytes within the NH Black category (*r* = 0.71), as well as markedly lower MAE for predictions of cell proportions compared to other epigenetic predictors. Performance of plasma protein predictors varied more widely by race/ethnicity. Correlation of the B2M predictor was highest in the NH White group (*r* = 0.56) and lowest in the Other/Multiracial group (*r* = 0.31), correlation of the CRP predictor was highest in the NH White group (*r* = 0.41) and lowest in the NH Black group (*r* = 0.28), correlation of the Cystatin C predictor was highest in the NH White group (*r* = 0.50) and lowest in the Other/Multiracial group (*r* = 0.22), and correlation of the HbA1c predictor was highest in the Mexican American group (*r* = 0.48) and lowest in the Other Hispanic group (*r* = 0.26). Furthermore, the CRP predictor also exhibited markedly higher MAE compared to the other plasma protein and cell proportion predictors, ranging from 1.16 mg/L in the Other/Multiracial group to 2.05 mg/L in the NH Black group (Fig. S1). It is important to note that the original GrimAge manuscript, the source of the Cystatin C and B2M predictors, utilized a correlation threshold of 0.35 to judge performance of plasma protein predictors. In the race/ethnicity-stratified analysis, this 0.35 correlation threshold was only exceeded for both the B2M and Cystatin C predictors within the NH White and Other Hispanic groups. Correlation and MAE for each predictor is presented in Table S3. Complete scatterplots displaying the fit between epigenetic predictions and corresponding laboratory-derived variable or chronological age for each predictor can be found in Fig. S2.

### Bootstrapped differences in performance

**Fig. 2:**
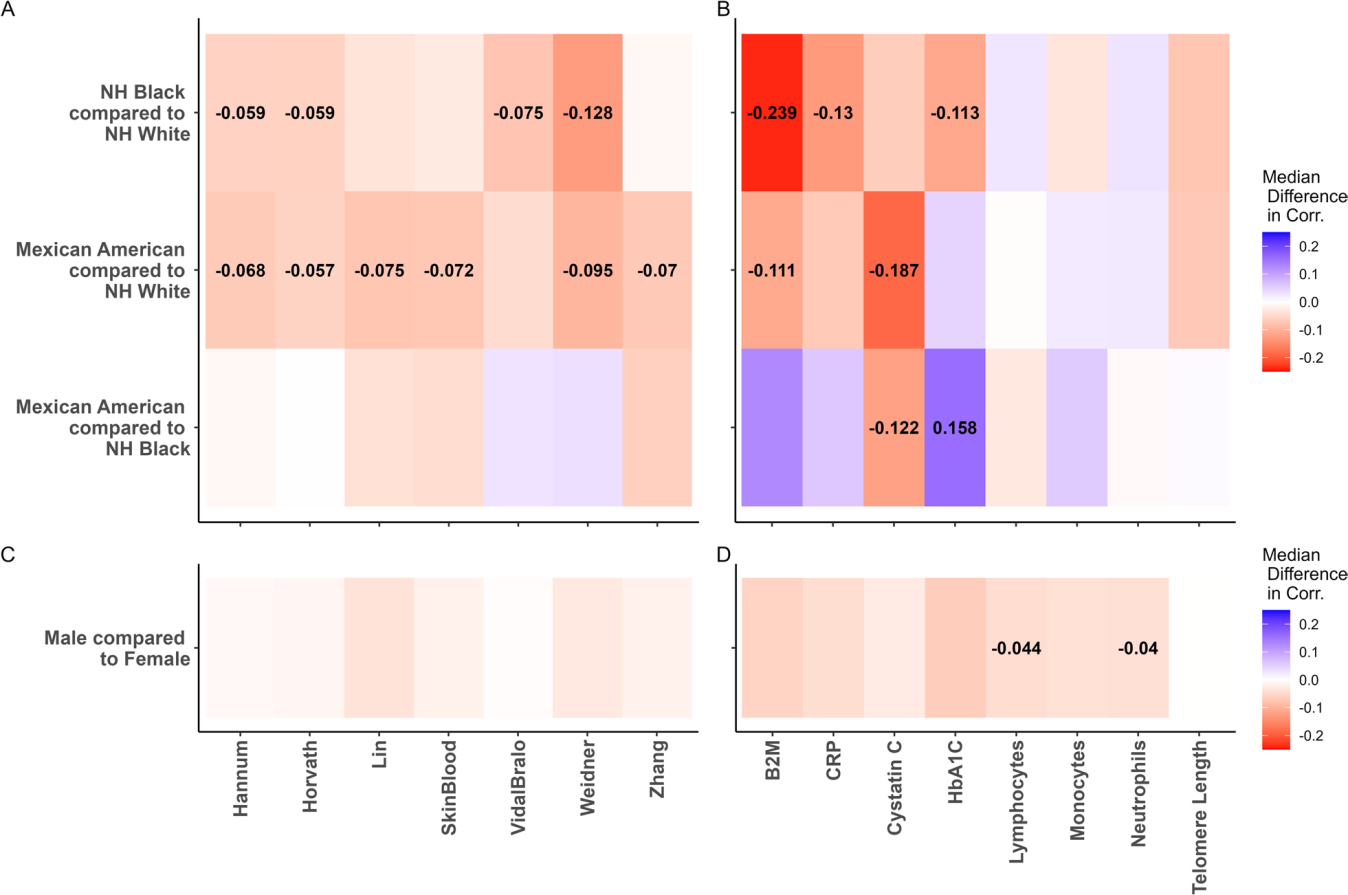
Bootstrapped median differences in Pearson Correlation coefficients for epigenetic predictors, stratified by race/ethnicity category (**A**, **B**) and stratified by sex (**C**, **D**). Color scale denotes median difference in correlation from 10,000 iterations of bootstrapping, and median difference in correlation is displayed for significant differences

Bootstrapped differences in correlation coefficients between NH White, NH Black, and Mexican American groups are presented in Fig. 2A, B, and differences in correlation coefficients between males and females are presented in Fig. 2C, D. Most epigenetic clocks followed a general trend of lower correlation with chronological age in the NH Black group compared to the NH White group, lower correlation in the Mexican American group compared to the NH White group, and largely null differences in correlation between the Mexican American and NH Black groups. These differences were especially pronounced with the Weidner clock, with significantly lower correlation in the Mexican American group compared to the NH White group (Median difference in correlation: − 0.10, 95% CI − 0.17, − 0.02) and significantly lower correlation in the NH Black group compared to the NH White group (Median difference in correlation: − 0.13, 95% CI − 0.21, − 0.05), as well as with the Hannum clock, with significantly lower correlation in the Mexican American group compared to the NH White group (Median difference in correlation: − 0.07, 95% CI − 0.14, − 0.01) and significantly lower correlation in the NH Black group compared to the NH White group (Median difference in correlation: − 0.06, 95% CI − 0.10, − 0.02).

Bootstrapping also revealed significant differences between groups for several plasma protein predictors. In particular, the B2M predictor exhibited lower correlation in Mexican Americans compared to the NH White group (Median difference in correlation: − 0.11, 95% CI − 0.21, − 0.01) and significantly lower correlation in the NH Black group compared to the NH White group (Median difference in correlation: − 0.24, 95% CI − 0.34, − 0.14). The Cystatin C predictor also exhibited significantly lower correlation in Mexican Americans compared to the NH White group (Median difference in correlation: − 0.19, 95% CI − 0.28, − 0.10) and significantly lower correlation in the Mexican American group compared to the NH Black group (Median difference in correlation: − 0.12, 95% CI − 0.23, − 0.01). Additionally, the HbA1c predictor exhibited significantly higher correlation in the Mexican American group compared to the NH Black group (Median difference in correlation: 0.16, 95% CI 0.05, 0.27) and significantly lower correlation in the NH Black group compared to the NH White group (Median difference in correlation: − 0.11, 95% CI − 0.22, − 0.01).

In contrast to frequent differences in correlation observed in relation to race and ethnicity categories, there was less evidence for significant differences in performance of predictors between males and females, with the only significant differences indicating lower correlations among males compared to females for lymphocytes (Median difference in correlation: − 0.04, 95% CI − 0.08, − 0.01) and neutrophils (Median difference in correlation: − 0.04, 95% CI − 0.07, − 0.01). Interestingly, these lower correlations with laboratory-derived cell proportions among males were observed despite males making up 84% of the cell proportion training sample.

**Fig. 3:**
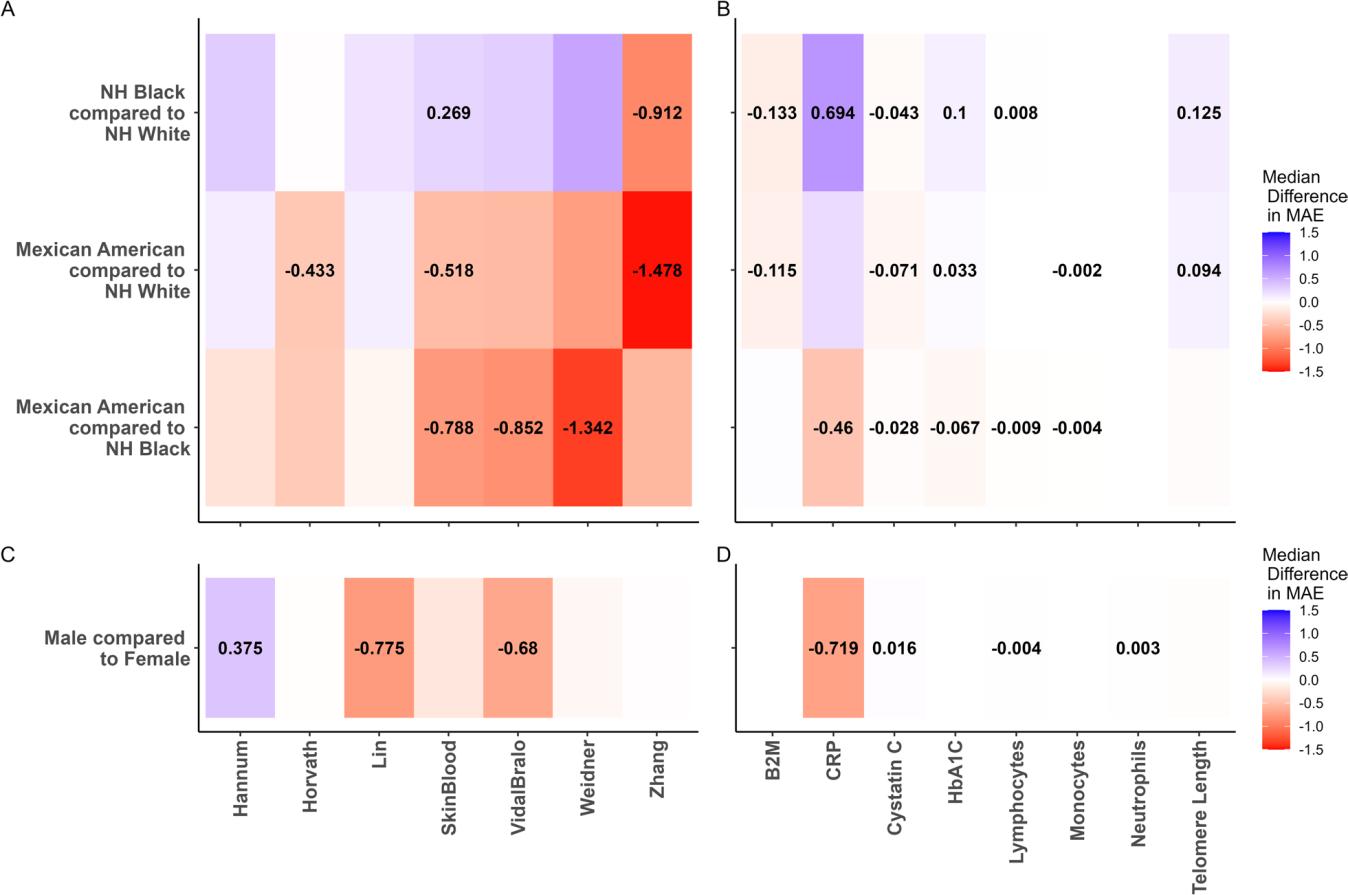
Bootstrapped median differences in MAE for epigenetic predictors, stratified by race/ethnicity category (**A**, **B**) and stratified by sex (**C**, **D**). Color scale denotes median difference in MAE from 10,000 iterations of bootstrapping, and median difference in MAE is displayed for significant differences

Bootstrapping analysis also revealed substantial evidence for significant differences in MAE between groups across most predictors, however, there was relatively more variability of trends in differences between groups (Fig. 3). Race and ethnicity-related differences in MAE for the epigenetic clocks were especially pronounced with the Zhang clock, with lower MAE in Mexican Americans compared to the NH White group (Median difference in MAE: − 1.48, 95% CI − 1.91,  − 0.98) and lower MAE in the NH Black group compared to the NH White group (Median difference in MAE: − 0.91, 95% CI − 1.49,  − 0.41), as well as for the Skin&Blood clock; with lower MAE in Mexican Americans compared to the NH Black group (Median difference in MAE:  − 0.79, 95% CI − 1.17, − 0.47), lower MAE in Mexican Americans compared to the NH White group (Median difference in MAE:  − 0.52, 95% CI − 0.82,  − 0.21), and higher MAE in the NH Black group compared to the NH White group (Median difference in MAE: 0.27, 95% CI 0.001, 0.62). The most pronounced differences for the plasma protein predictors were observed with CRP, with lower MAE in Mexican Americans compared to the NH Black group (Median difference in MAE: − 0.46, 95% CI − 0.86, − 0.08) and higher MAE in the NH Black group compared to the NH White group (Median difference in MAE: 0.69, 95% CI 0.33, 1.08). Additionally, both the Cystatin C and HbA1c predictors exhibited significant differences in MAE across all three comparisons between Race/Ethnicity groups. MAE for the telomere length predictor was significantly higher in the NH Black group compared to the NH White group (Median difference in MAE: 0.13, 95% CI 0.07, 0.19), as well as significantly higher in the Mexican American group compared to the NH White group (Median difference in MAE: 0.09, 95% CI 0.05, 0.14). Additionally, we observed several low-magnitude significant differences in MAE observed for the cell proportion predictors, including higher MAE for the lymphocyte proportion estimates in the NH Black group compared to the NH White group (Median difference in MAE: 0.008, 95% CI 0.004, 0.013) and significantly lower MAE in the Mexican American group compared to the NH Black group (Median difference in MAE: − 0.009, 95% CI − 0.014, − 0.005).

Additionally, there was evidence for significant sex-related differences in MAE across several predictor including lower MAE for the Lin clock in males (Median difference in MAE: − 0.78, 95% CI − 1.46, − 0.06), lower MAE for the Vidal-Bralo clock in males (Median difference in MAE: − 0.68, 95% CI − 1.20, − 0.07), higher MAE for the Hannum clock in males (Median difference in MAE: 0.38, 95% CI 0.05, 0.67), lower MAE for the CRP predictor in males (Median difference in MAE: − 0.72, 95% CI − 0.96, − 0.51), and higher MAE for the neutrophil proportion predictor in males (Median difference in MAE: 0.003, 95% CI 0.001, 0.006). Complete summaries for differences in correlation and MAE for each predictor are presented in Table S4.

As a sensitivity analysis, we further examined the potential influence of differences in sample sizes between groups on our findings by repeating the bootstrap analysis after limiting sample sizes for comparisons to the smallest group’s sample size (Figs. S3 and S4). The observed trends closely resembled those from the primary analysis, albeit with modestly larger confidence intervals for some comparisons, indicating that differences in sample sizes between groups are not driving observed associations. Complete summaries for differences in correlation and MAE for each predictor from this sensitivity analysis are presented in Table S5.

### Regression analyses

**Fig. 4:**
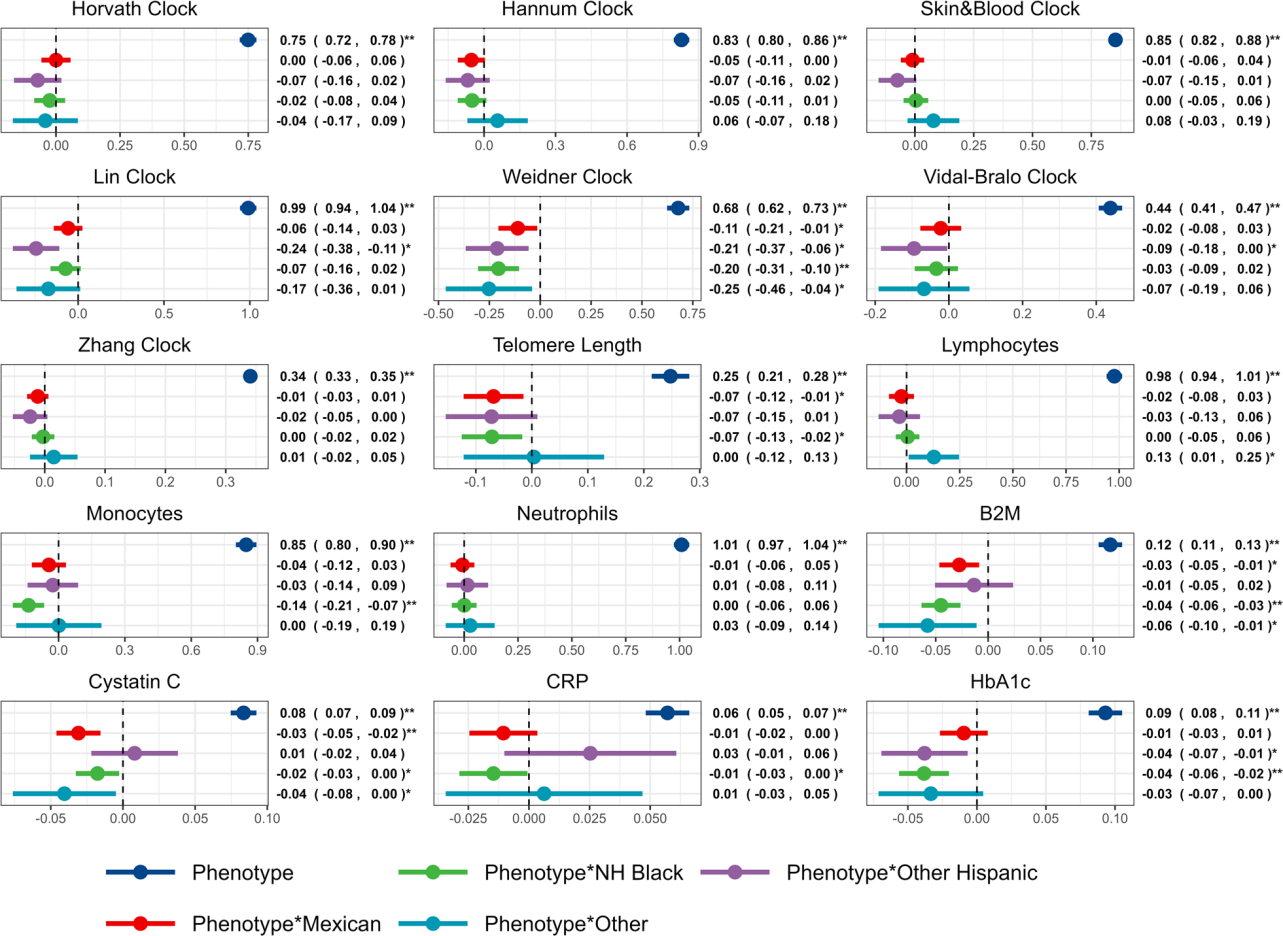
Effect estimates and 95% CIs from multivariate linear regression model summaries (minimally-adjusted) including the epigenetic prediction as the outcome; the phenotypic trait, race/ethnicity, an interaction term between race/ethnicity and the phenotypic trait as the predictor variables further adjusted for sex. Phenotype effect estimates (dark blue) reflect the expected change in epigenetic prediction for a 1 unit change in the phenotypic trait within the reference group (NH White participants). The interaction terms represent the additional expected change in the association between the phenotype and the epigenetic prediction within the specified group. “**” indicates a Bonferroni-adjusted *p*-value < 0.05. “*” indicates an unadjusted *p*-value < 0.05

Results from the regression analyses further reinforced findings from the bootstrap analysis. Each of the phenotypic traits were strongly associated with the epigenetic prediction in the reference groups (NH White participants for the race/ethnicity analysis and female participants in the sex analysis), with regression coefficients ranging from 0.06 with CRP in the race/ethnicity models to 1.04 for the neutrophil predictor in the sex models (Table S6). The interaction terms in 6 models met the stringent multiple testing threshold. These interactions suggested decreased associations between the phenotypic trait and the epigenetic prediction among NH Black participants compared to NH White participants for the B2M predictor (beta = − 0.04, 95% CI − 0.06, − 0.03), HbA1c predictor (beta = − 0.04, 95% CI − 0.06, − 0.02), the Weidner clock (beta = − 0.20, 95% CI − 0.31, − 0.10), and the monocyte proportion predictor (beta = − 0.14, 95% CI − 0.21, − 0.07), as well as a decreased association for the Cystatin C predictor among Mexican American participants compared to NH White participants (beta = − 0.03, 95% CI − 0.05, − 0.02) (Fig. 4). There was also evidence for a decreased association for the lymphocyte predictor among males compared to females (beta = − 0.09, 95% CI − 0.13, − 0.05) (Fig. S5). An additional 16 interaction terms featured unadjusted *p*-values < 0.05, with most suggesting decreased associations between the phenotypic trait and the epigenetic predictor related to race/ethnicity.

Finally, we excluded the cell proportion estimators as variables of interest and examined whether further adjusting for SES-related variables and DNAm-derived estimates of cell proportions would influence our findings. All four associations remained significant, indicating decreased associations between the phenotypic trait and the epigenetic predictor among NH Black participants compared to NH White participants for B2M predictor (beta = − 0.04, 95% CI − 0.05, − 0.02), HbA1c predictor (beta = − 0.04, 95% CI − 0.05, − 0.02), and the Weidner clock (beta = − 0.23, 95% CI − 0.33, − 0.12), as well as decreased associations among Mexican American participants compared to NH White participants for the Cystatin C predictor (beta = − 0.03, 95% CI − 0.04, − 0.01) (Figs. S6-S7). Similarly, an additional 14 interaction terms featured unadjusted *p*-values < 0.05, with most suggesting decreased associations between the phenotypic trait and the epigenetic predictor related to race/ethnicity (Table S7). Taken together, these results suggest that disparities in predictor performance are present after accounting for overall differences in epigenetic predictions related to race/ethnicity and sex, as well as after further adjustment for estimated cell proportions and SES variables.

## Discussion

We performed an extensive evaluation of the performance of epigenetic predictors of chronological age, telomere length, circulating plasma protein levels, and leukocyte proportions in a large sample of US adults. Although most epigenetic predictors performed well in the NHANES population as a whole, we found evidence for substantial variation in performance by self-reported race/ethnicity, both in terms of correlation and MAE, as well as some evidence for variation in performance by sex. Each of the included epigenetic predictors exhibited at least one significant difference in correlation or MAE in relation to race/ethnicity or sex, highlighting the pervasiveness of this issue. We observed a reoccurring pattern of lower correlation among NH Black and Mexican American participants compared to NH White participants across most epigenetic clocks and predictors of plasma protein levels. Regression analysis further supported this finding, indicating that disparities in epigenetic predictor performance persist after accounting for overall differences in epigenetic predictions related to race/ethnicity and sex, as well as further adjustment for estimated cell proportions and SES variables. Differences in MAE between groups tended to vary more widely between predictors with less evidence for overarching patterns.

Some of the highest variability in correlation between race/ethnicity groups was observed with the GrimAge plasma protein predictors as well as the Weidner and Hannum clocks, with reoccurring trends of lower correlations among Mexican American and NH Black participants compared to NH White participants. The GrimAge predictors and Hannum clock were trained in majority White populations, while the Weidner clock was trained in a sample of mostly unspecified racial/ethnic background. Relatively small differences in performance of epigenetic predictors can have substantial consequences. For example, the original GrimAge manuscript, the source of the Cystatin C and B2M predictors used in our study, utilized a minimum correlation threshold of 0.35 in both training and testing datasets as a minimum performance metric to select component predictors for the final GrimAge model [5]. However, both the training and testing datasets were composed of participants from the Framingham Heart Study, a predominately White study sample. In the NHANES population, this 0.35 correlation threshold was only exceeded for both the B2M and Cystatin C predictors within the NH White and Other Hispanic groups, suggesting that these predictors would be less likely to pass the pre-specified threshold if the testing samples were drawn from more diverse populations.

Interpretation of our findings requires a careful delineation between signal and noise related to race. Race is a social construct that is often used in public health research as a proxy for observed disparities in social and environmental exposures related to systemic racism [37]. Accordingly, a wide range of studies have reported associations between race and epigenetic aging, with studies based in the US often reporting higher epigenetic age acceleration (EAA) in Black populations compared to White populations, albeit with substantial heterogeneity in the strength of associations between different populations and for different epigenetic clocks [9, 10, 38–40]. These observed associations are likely related to disparities in several factors including systemic and structural racism, socioeconomic status (SES), healthcare access and utilization, and exposure to environmental pollution. Accordingly, several studies have begun to dissect the association between race and EAA to better understand the specific factors driving these observed associations. For example, an analysis within the Health and Retirement Study found that Black participants had higher average EAA compared to White participants, and large portions of this association was explained by disparities in individual level SES and neighborhood deprivation [41]. Similarly, a study within the Future of Families and Child Well-Being study found higher EAA among Black adolescents compared to their White counterparts, with greater exposure to police intrusion playing a role in explaining this higher EAA [40]. It is also important to highlight that these findings reflect social structures present in the US, with distinct trends likely to be present in other regions of the world [42].

While the preceding examples provide evidence for signal related to race for epigenetic biomarkers, our analysis was more concerned with noise related to race surrounding epigenetic predictions. Key demographic variables including race, ethnicity, country of origin, and sex tend to be poorly reported in epigenetic clock training datasets, and White/European populations tend to be overrepresented in epigenetic predictor training datasets [13]. This issue of training sample diversity and subsequent limited generalizability to external populations extends well beyond epigenetic predictors, with similar issues raised in the development of polygenic risk scores and predictive models based on electronic health records [14–16]. Low diversity of training samples of epigenetic predictors may lead to poor performance when applied to external populations distinct from the populations represented in the training samples, with potential further downstream consequences related to the interpretation of stratified analyses, where subgroup-specific effect estimates may be influenced by varying levels of noise surrounding epigenetic predictions within those subgroups. However, limited research has explicitly sought to examine the performance of epigenetic predictors in relation to race and ethnicity. Similar to the results of our study, a previous analysis in the ECHO consortium found variable performance of several pediatric epigenetic clocks between Black and White children [11].

The underlying causes of differences in epigenetic predictor performance between populations are also not well understood. Epigenetic predictors are trained to learn patterns of association between DNAm and phenotypes of interest. However, DNAm can vary widely in response to a number of factors, and predictors are ultimately limited to learning patterns that are present within the original training dataset. One key source of DNAm variability is genetics, where genetic variation linked to ancestry has been shown to have a profound influence on DNAm variability between populations [43, 44]. Furthermore, DNAm can be highly responsive to environmental exposures and health status, and racial disparities in socioeconomic status, exposure to environmental pollutants, and health outcomes in the US are pervasive and well documented [45–47]. Taken together, poorer performance of epigenetic predictors in external populations may represent the learning of patterns that are overly specific to the training sample, or may be related to limited variability in a combination of genetic, social, and environmental factors in the training sample. Critically, we found that observed disparities in epigenetic predictor performance persisted after accounting for overall differences in epigenetic predictions related to race/ethnicity and sex, variability in key SES measures, and estimated cell proportions.

We observed relatively distinct trends in differences in predictor performance between groups depending on whether correlation coefficients or MAE were used as the performance metric. Pearson correlation coefficients capture the strength of the linear relationship between two variables while MAE captures the median difference between two variables, providing two complementary but distinct performance metrics, where higher correlation and lower MAE are seen as beneficial. We found several cases where comparisons between groups led to agreement in directionality of performance metrics, including lower correlation and higher MAE for the CRP predictor in the NH Black group compared to the NH White group. However, we also observed several cases of diverging estimates of performance between correlation and MAE, including lower correlation and lower MAE for the Zhang clock when comparing the Mexican American group to the NH White group. The Zhang clock featured the highest correlation among all considered epigenetic clocks, but also featured a relatively high MAE, stemming from systematic overestimation of age in younger participants and underestimation of age in older participants. Although the linear correlation between Zhang age estimates and chronological age was high, MAE was relatively high and varied more substantially between groups, highlighting the importance of using varying performance metrics to evaluate predictor performance.

Our study features several key strengths. Firstly, by harnessing the extensive selection of laboratory measures available through NHANES, we were able to compare the performance of a wide range of epigenetic predictors including predictors of age, telomere length, plasma protein levels, and cell proportions. Secondly, the NHANES sample features a large and diverse population which enabled detailed race/ethnicity- and sex-stratified analysis. Third, in our regression analysis we found that disparities in epigenetic predictor performance persisted after accounting for overall differences in epigenetic predictions related to race/ethnicity and sex, as well as estimated cell proportions and SES variables. However, our findings are subject to several limitations. Firstly, we relied on the five category classification of race and ethnicity used by NHANES during the 1999–2000 and 2001–2002 survey cycles. These are socially-constructed categories which are inadequate representations of true human variation. Furthermore, our bootstrapping analysis was limited to comparisons between three groups (Mexican American, NH White, and NH Black) with sufficient sample sizes. Relatedly, we had no genetic data available to examine the influence of genetic ancestry within this context. We also cannot discount the potential role of genetic ancestry, or differing distributions of other environmental and lifestyle factors in driving observed differences between groups. Additionally, our analysis featured a large number of independent tests, inviting the possibility of false positives. To address this limitation, we included a stringent multiple testing adjustment in the linear regression analysis to identify high-confidence results. Finally, countless epigenetic predictors have been developed for a wide array of phenotypic traits, and our analysis was limited to the subset of epigenetic clocks and other predictors included in the public NHANES epigenetic clocks dataset with corresponding laboratory measures available.

## Conclusions

Epigenetic predictors are often developed utilizing convenience samples, harnessing data from public repositories or existing large cohort studies. However, because participants of European ancestry are historically overrepresented in epigenetics research, this convenience-based sampling may lead to limited diversity of populations used to train epigenetic predictors. We found that each epigenetic predictor exhibited at least one significant difference in performance related to race/ethnicity or sex, as well as consistently lower correlation among NH Black and Mexican American participants compared to NH White participants across most epigenetic clocks and plasma protein predictors. Researchers should aim to build large diverse training samples and openly report basic demographic information relating to their training samples in order to ensure external validity of future epigenetic predictors.

## Supplementary Information

Additional file 1.

Additional file 2.

## Data Availability

All data used for this analysis is publicly available from the NHANES website (https://www.cdc.gov/nchs/nhanes/index.htm). All code necessary to reproduce this analysis is available on GitHub (https://github.com/D-Khodasevich/NHANES_Evaluation).
