# Supplementary material for: Influence of race, ethnicity, and sex on the performance of epigenetic predictors of phenotypic traits
Source: Clin Epigenetics. 2025 Apr 9;17:59. doi: 10.1186/s13148-025-01864-6 (PMC11983795; doi:10.1186/s13148-025-01864-6)
Supplement: Supplementary file 1 — Additional file 1. [file 13148_2025_1864_MOESM1_ESM.docx]

**Influence of Race, Ethnicity, and Sex on the Performance of Epigenetic Predictors of Phenotypic Traits**

**Supplemental Materials**

Dennis Khodasevich^1^, Nicole Gladish^1^, Saher Daredia^2^, Anne K Bozack^1^, Hanyang Shen^1^, Jamaji C Nwanaji-Enwerem^3^, Belinda L Needham^4^, David H Rehkopf^1,5,6,7,8^, Andres Cardenas^1,7,*^

*Affiliations*
^1^ Department of Epidemiology and Population Health, Stanford University, Palo Alto, CA, USA
^2^ Division of Epidemiology, Berkeley Public Health, University of California, Berkeley, Berkeley, CA, USA
^3^ Department of Emergency Medicine and Center of Excellence in Environmental Toxicology, Perelman School of Medicine, University of Pennsylvania, Philadelphia, PA, USA
^4^ Department of Epidemiology, University of Michigan, Ann Arbor, Michigan, USA
^5^ Department of Health Policy, Stanford University, Palo Alto, CA, USA
^6^ Department of Medicine (Primary Care and Population Health), Stanford University, Palo Alto, CA, USA
^7^ Department of Pediatrics, Stanford University, Palo Alto, CA, USA
^8^ Department of Sociology, Stanford University, Palo Alto, CA, USA

**Supplemental Table Legends:**

***Supplemental Table 1****: Distributions of demographic variables within the NHANES study population stratified by Race/Ethnicity category. Mean (SD) reported for age, poverty-to-income ratio, plasma protein levels, cell proportions, and telomere length, and count (percentage) provided for education level, sex, and smoking activity.*

***Supplemental Table 2****: Demographic information on the training samples used to develop each of the included epigenetic predictors. For training datasets composed of multiple datasets, we break down demographics for each component dataset. Demographic information was obtained from accompanying public datasets or corresponding manuscripts when available, and is limited to the sample sizes, level of detail, and specific terminology provided in original sources.*

***Supplemental Table 3****: Pearson correlation coefficient (MAE) for each epigenetic predictor stratified by Race/Ethnicity category and sex.*

***Supplemental Table 4****: Median bootstrapped differences in Pearson correlation coefficients (2.5^th^ and 97.5^th^ percentiles) and MAE (2.5^th^ and 97.5^th^ percentiles) for each epigenetic predictor between Race/Ethnicity groups and between males/females.*

***Supplemental Table 5****: Median bootstrapped differences in Pearson correlation coefficients (2.5^th^ and 97.5^th^ percentiles) and MAE (2.5^th^ and 97.5^th^ percentiles) for each epigenetic predictor between Race/Ethnicity groups and between males/females from the limited sample size sensitivity models.*

***Supplemental Table 6****: Multivariate linear regression model summaries (minimally-adjusted). Race/ethnicity models included the epigenetic prediction as the outcome; the phenotypic trait, race/ethnicity, an interaction term between race/ethnicity and the phenotypic trait as the predictor variables, further adjusted for sex. Sex models included the epigenetic prediction as the outcome; the phenotypic trait, sex, an interaction term between sex and the phenotypic trait as the predictor variables, further adjusted for race/ethnicity. NH White participants were encoded as the reference group for the race/ethnicity analysis and female participants were encoded as the reference group in the sex analysis. Phenotype effect estimates reflect the expected change in epigenetic prediction for a 1 unit change in the phenotypic trait within the reference group. The interaction term represents the additional expected change in the association between the phenotype and the epigenetic prediction within the specified group. “**” indicates a Bonferroni-adjusted p-value < 0.05. “*” indicates an unadjusted p-value < 0.05.*

***Supplemental Table 7****: Multivariate linear regression model summaries (fully-adjusted). Race/ethnicity models included the epigenetic prediction as the outcome; the phenotypic trait, race/ethnicity, an interaction term between race/ethnicity and the phenotypic trait as the predictor variables, further adjusted for sex, estimated cell proportions, poverty-to-income ratio, and education. Sex models included the epigenetic prediction as the outcome; the phenotypic trait, sex, an interaction term between sex and the phenotypic trait as the predictor variables, further adjusted for race/ethnicity, estimated cell proportions, poverty-to-income ratio, and education. NH White participants were encoded as the reference group for the race/ethnicity analysis and female participants were encoded as the reference group in the sex analysis. “**” indicates a Bonferroni-adjusted p-value < 0.05. “*” indicates an unadjusted p-value < 0.05.*

**Supplemental Figures:**


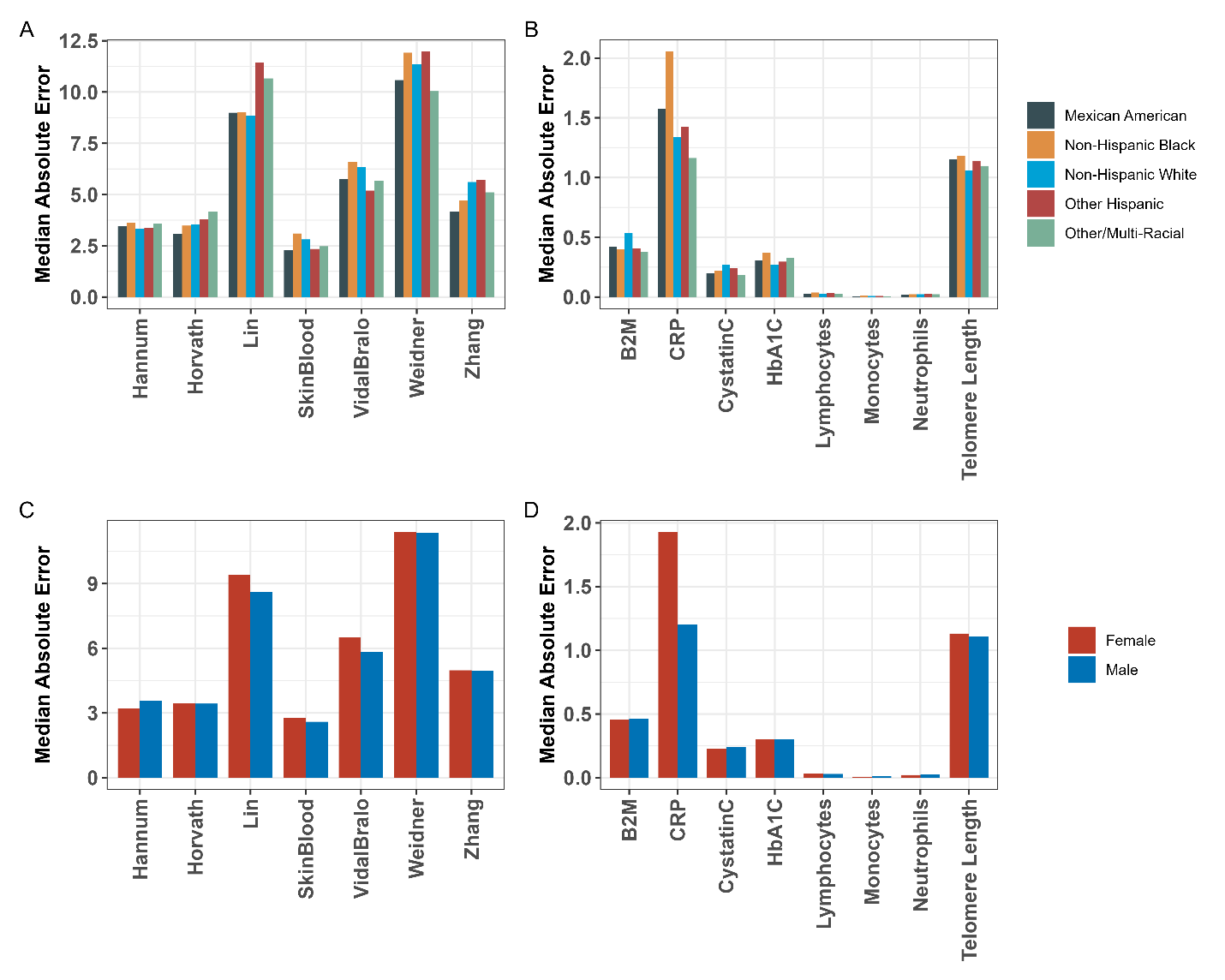


***Supplemental Figure 1****: MAE for epigenetic predictors, stratified by race/ethnicity category (A-B) and stratified by sex (C-D).*

***
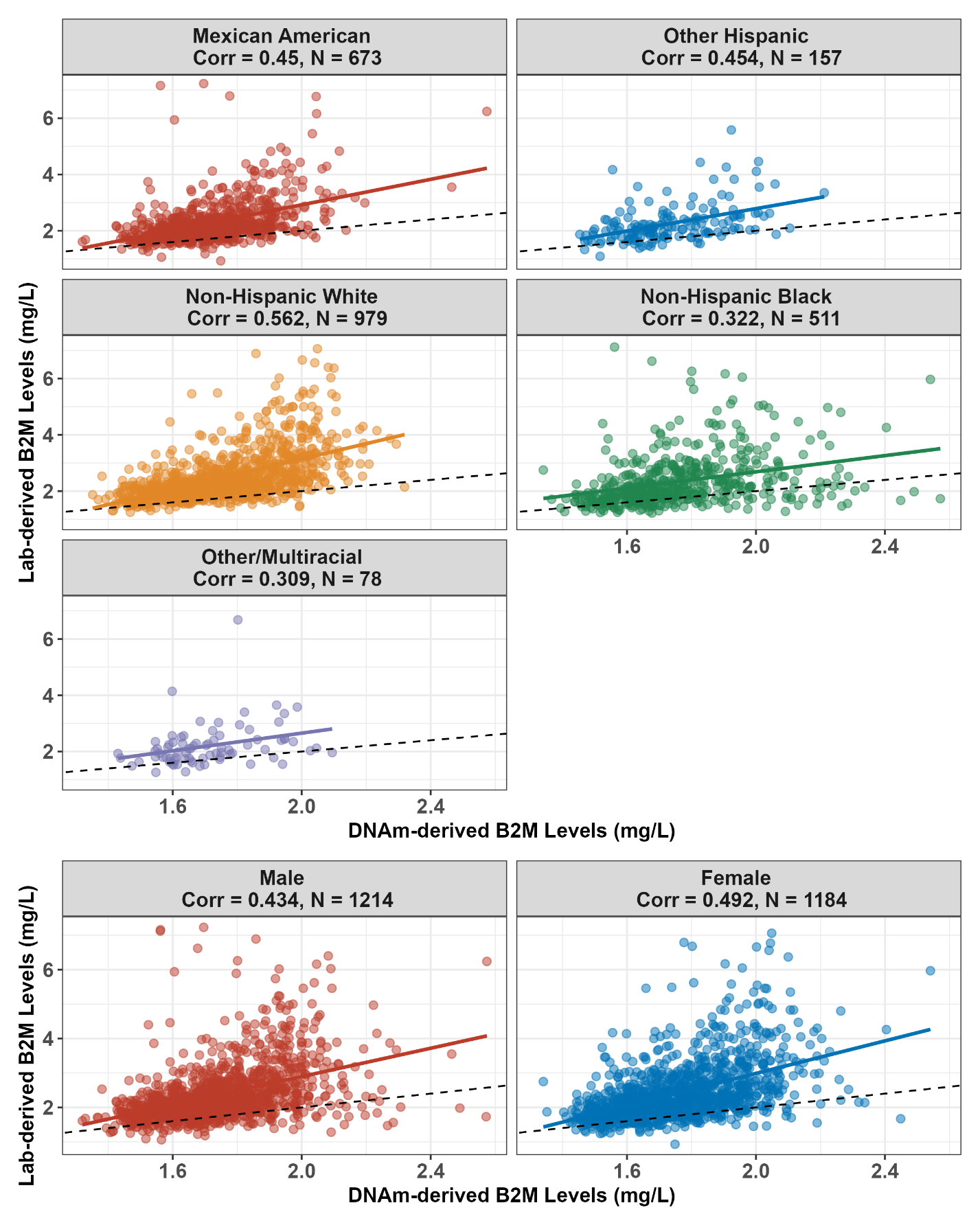
***

***
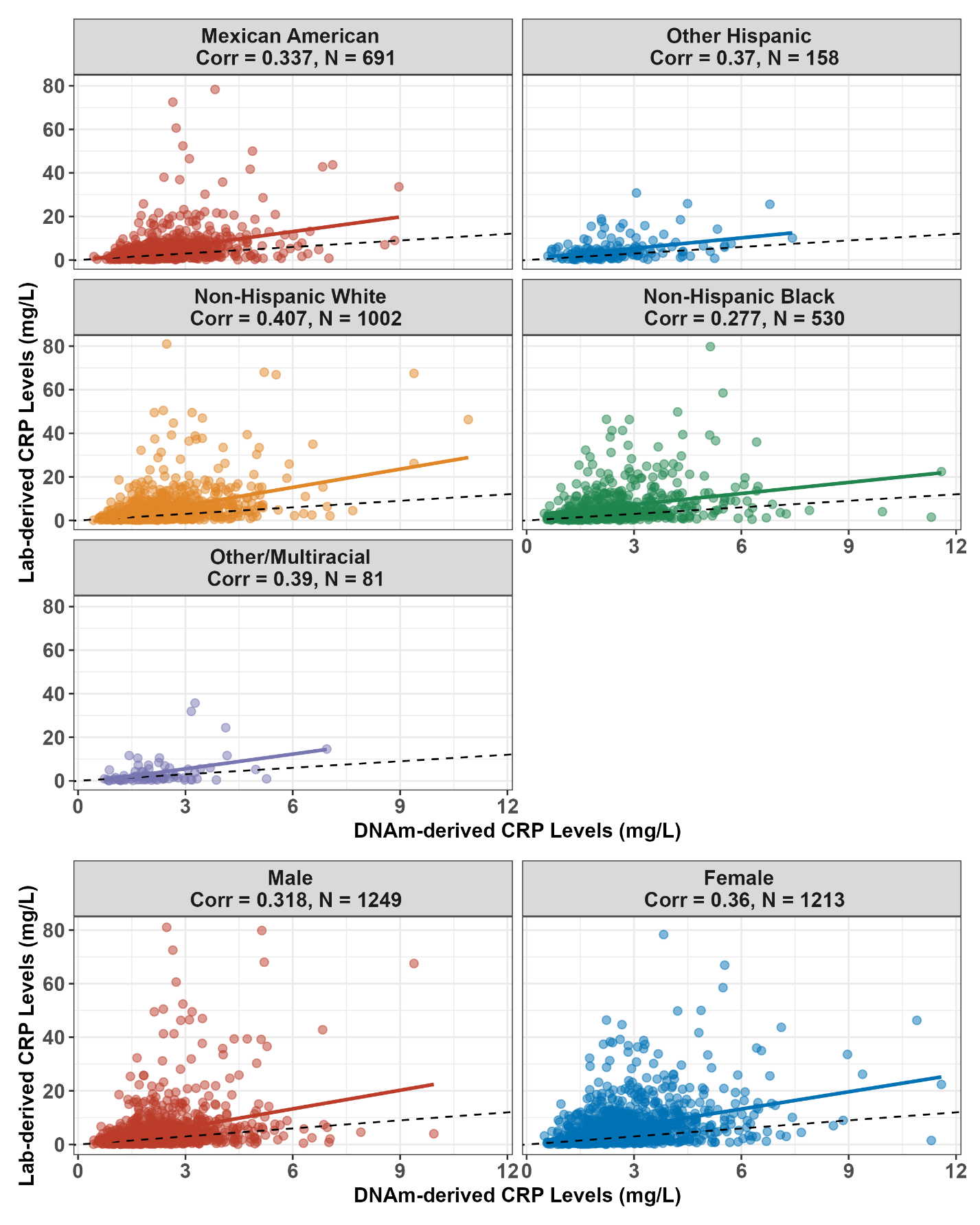
***

***
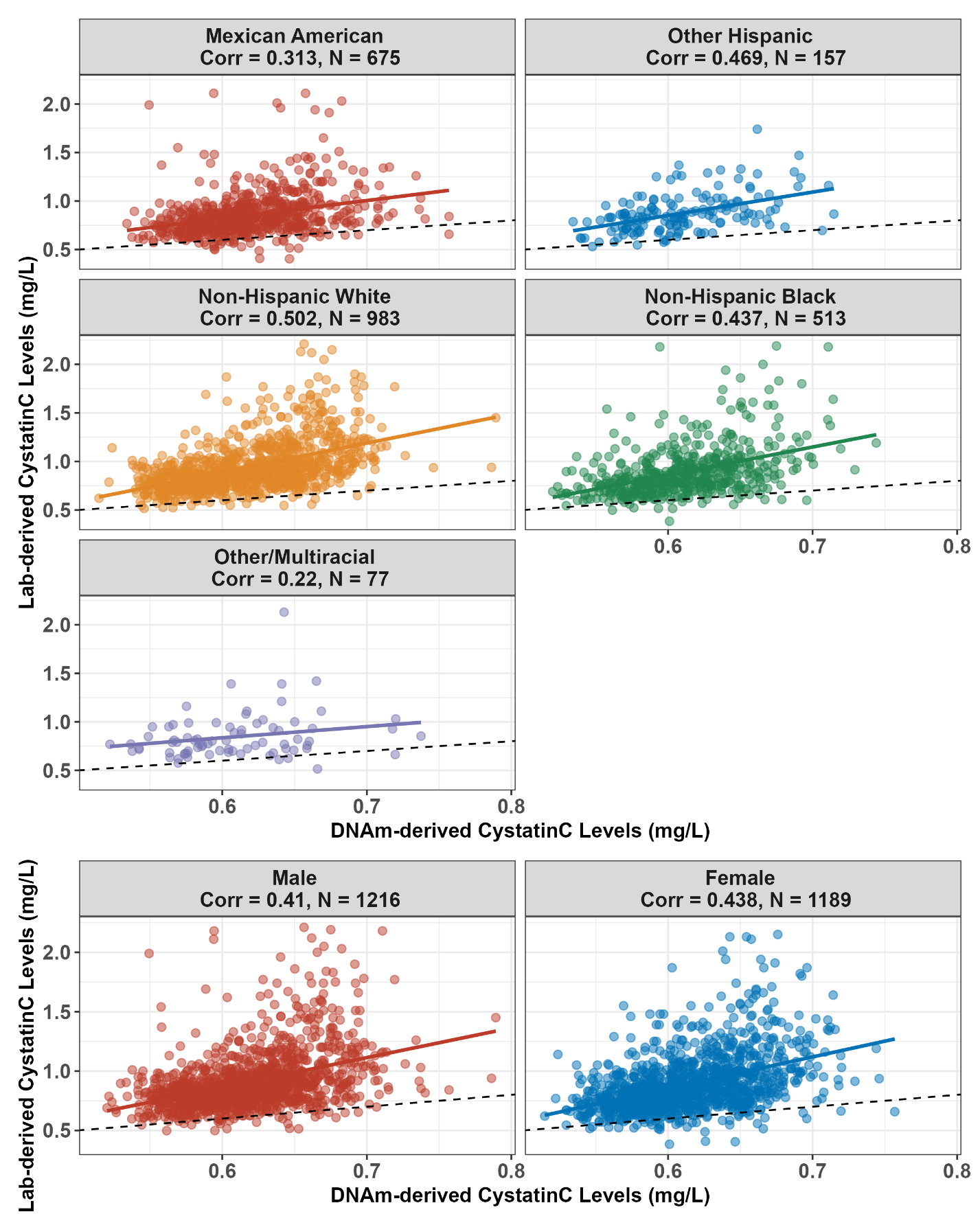
***

***
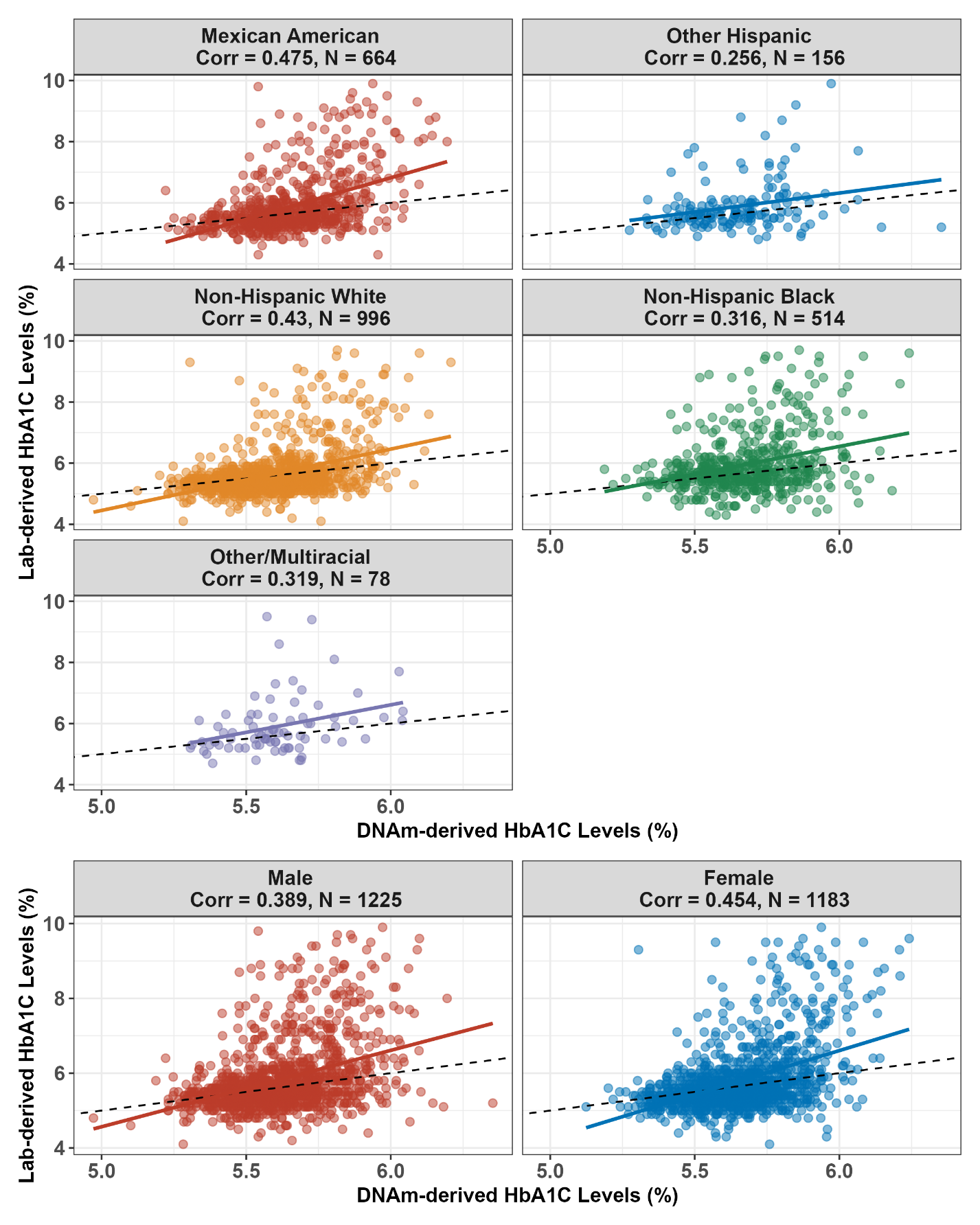
***

***
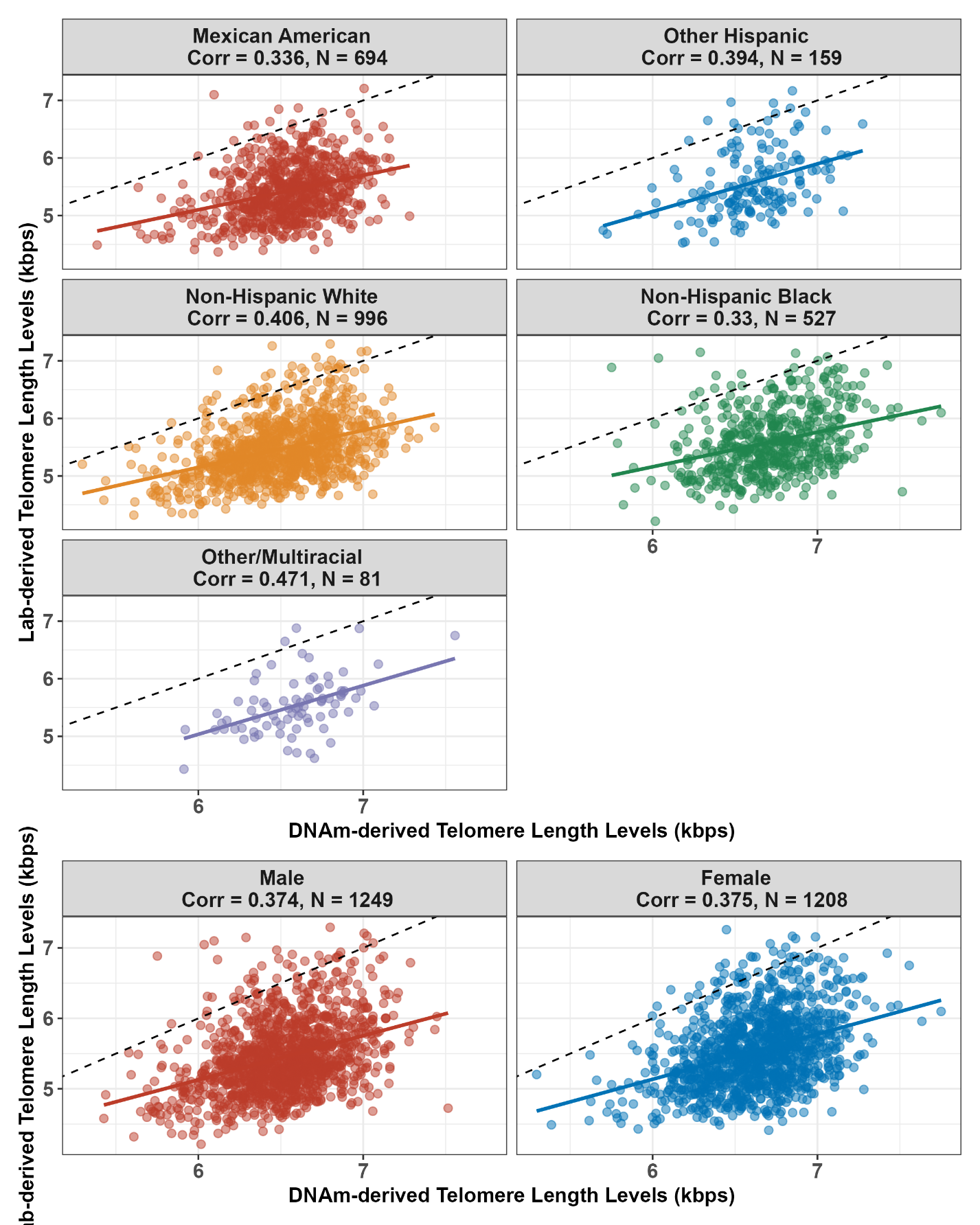
***

***
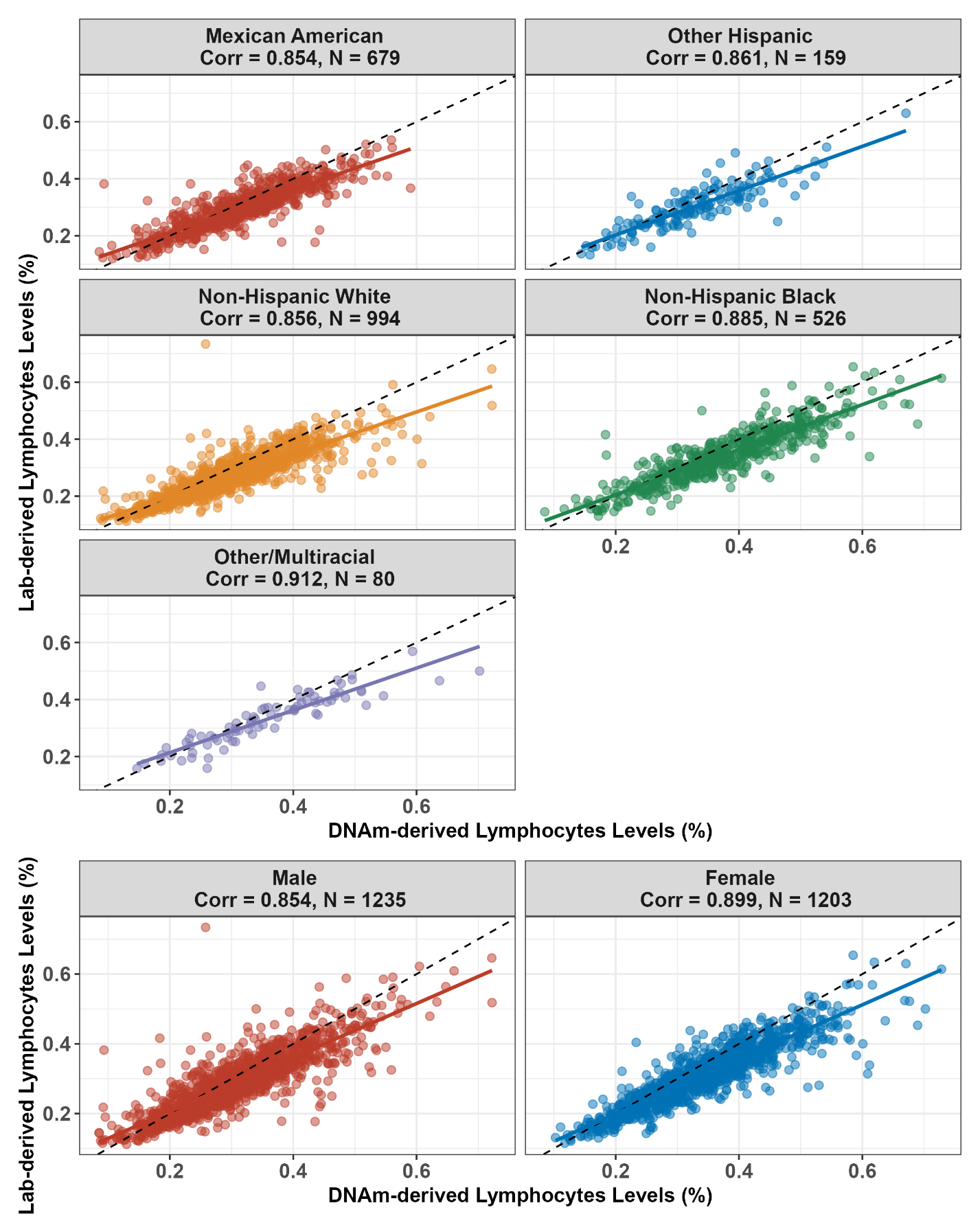
***

***
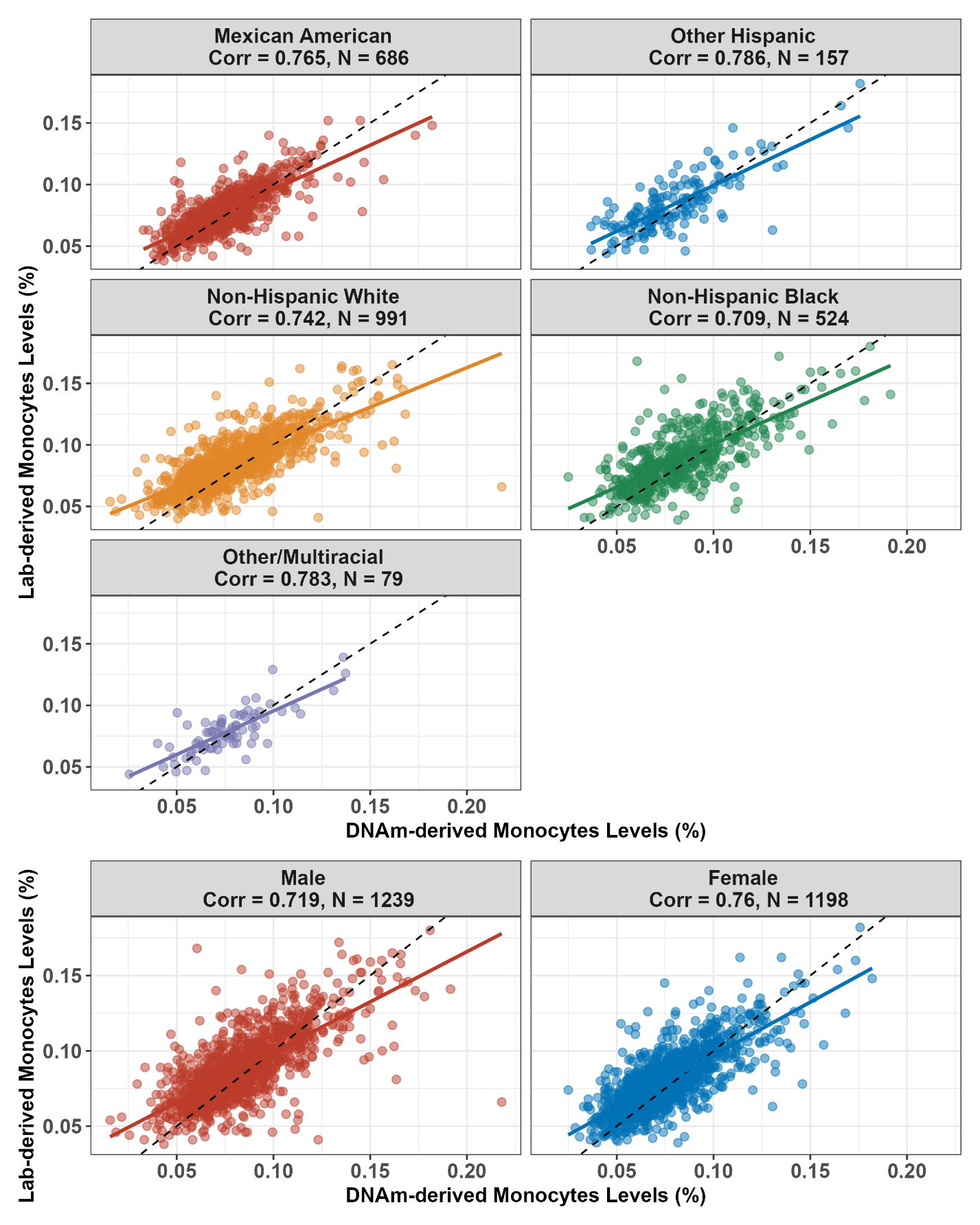
***

***
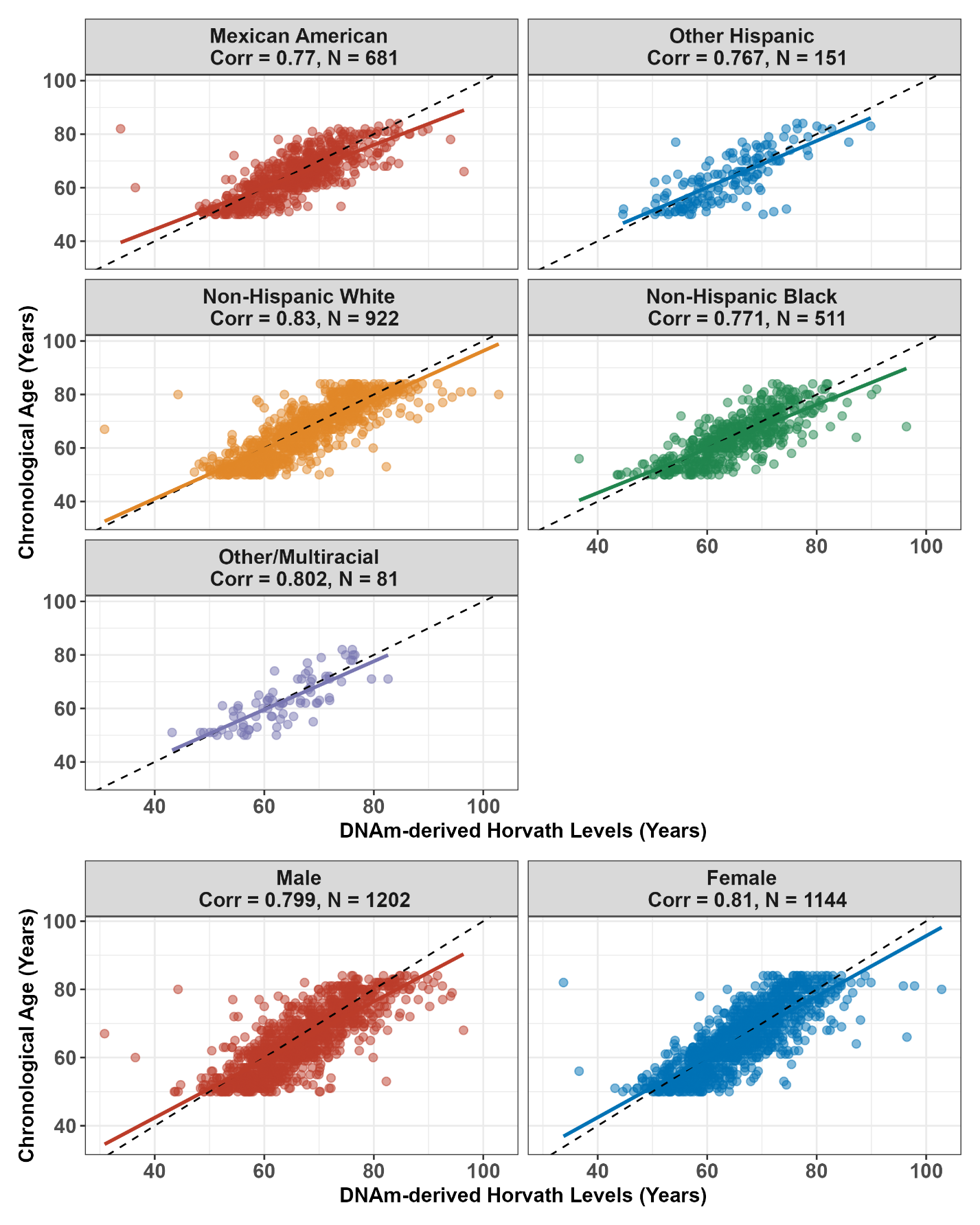

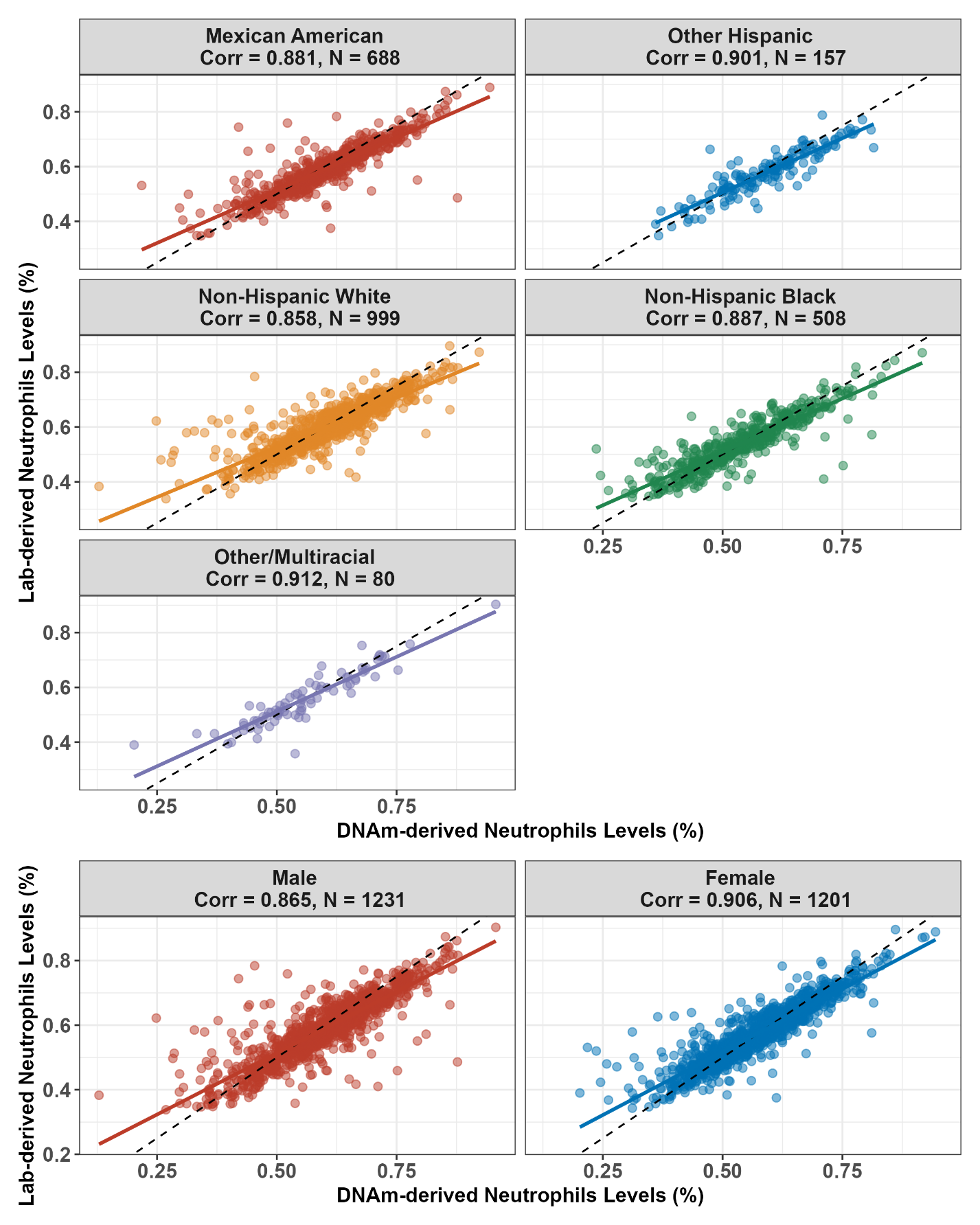
***

***
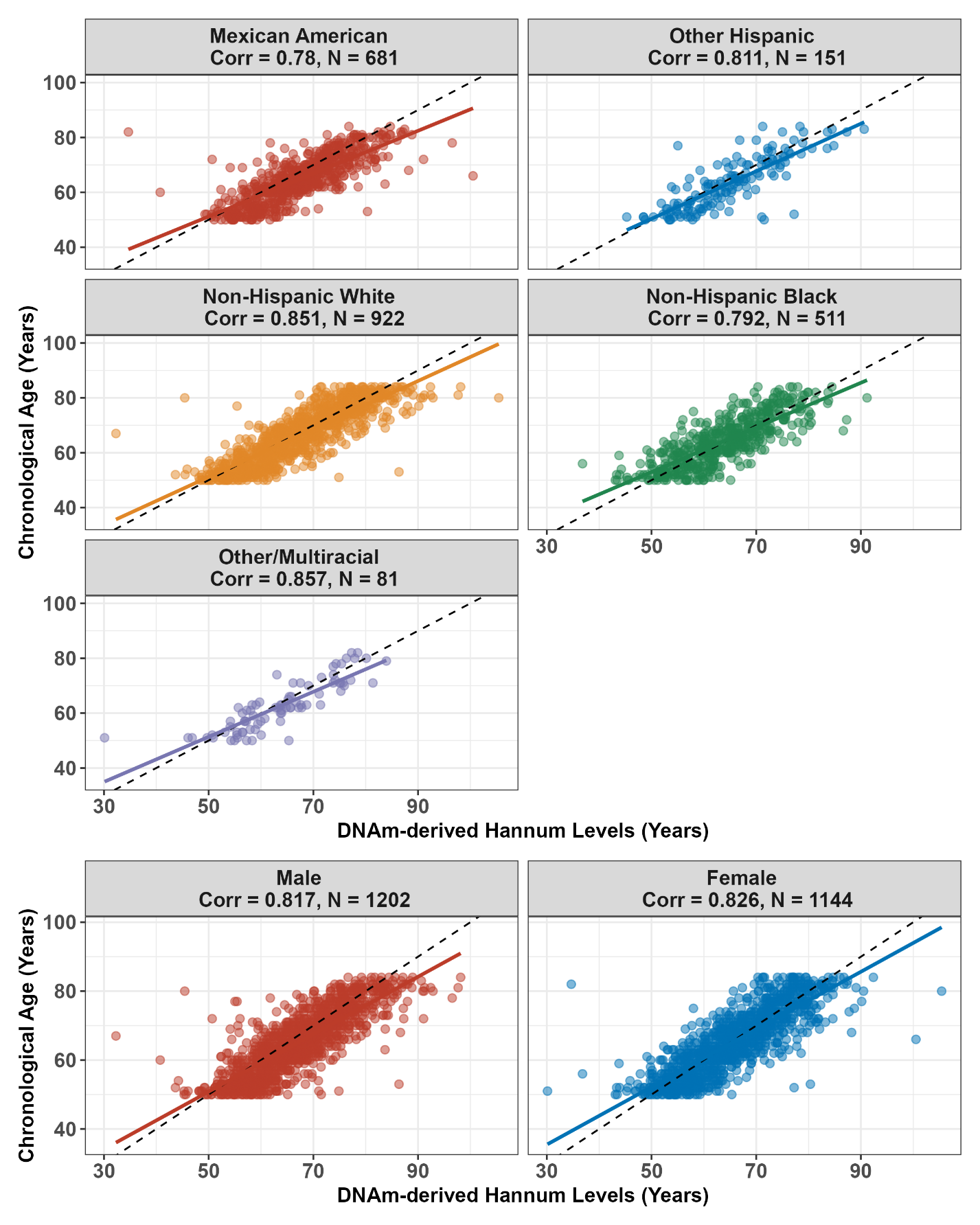
***

***
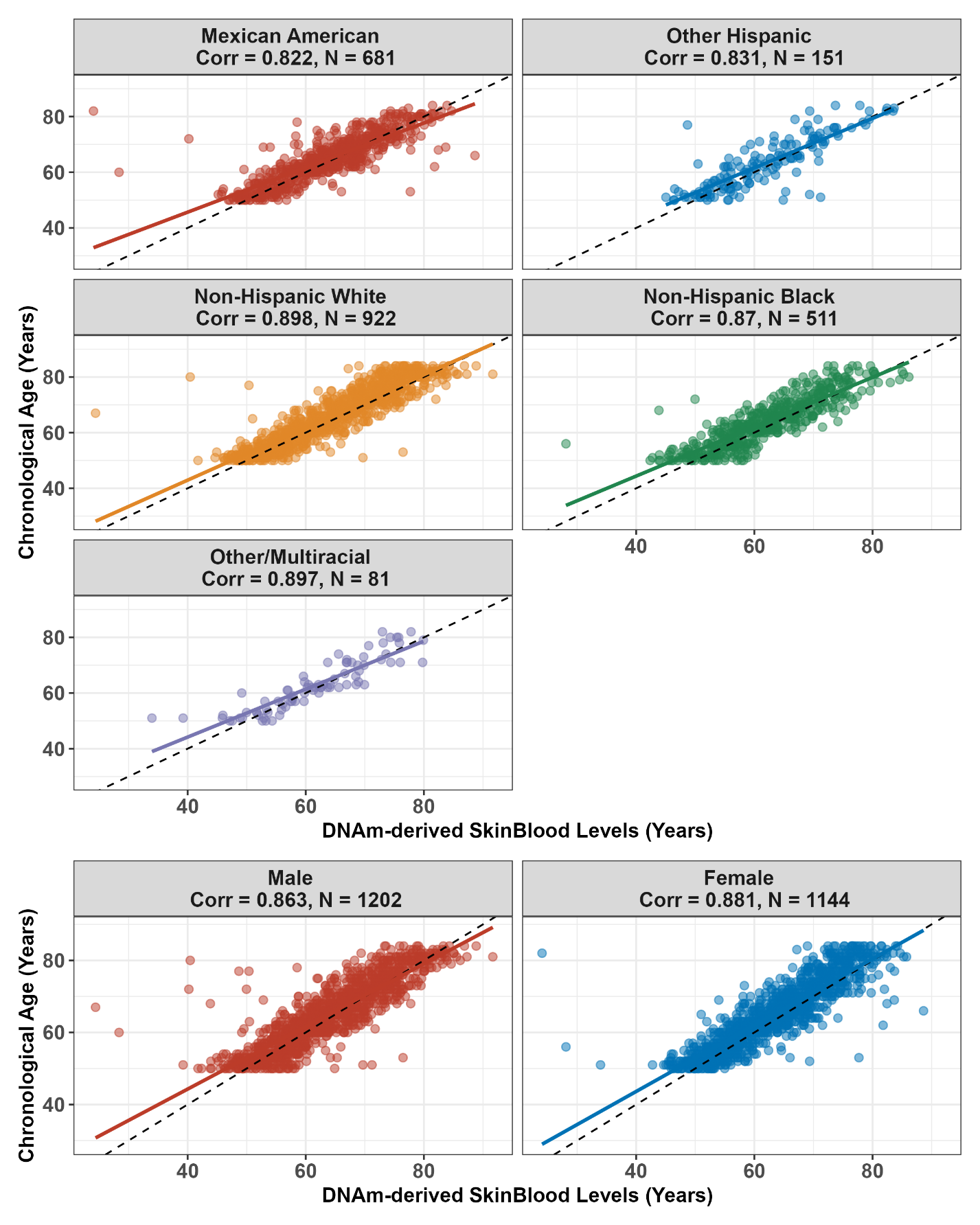
***

***
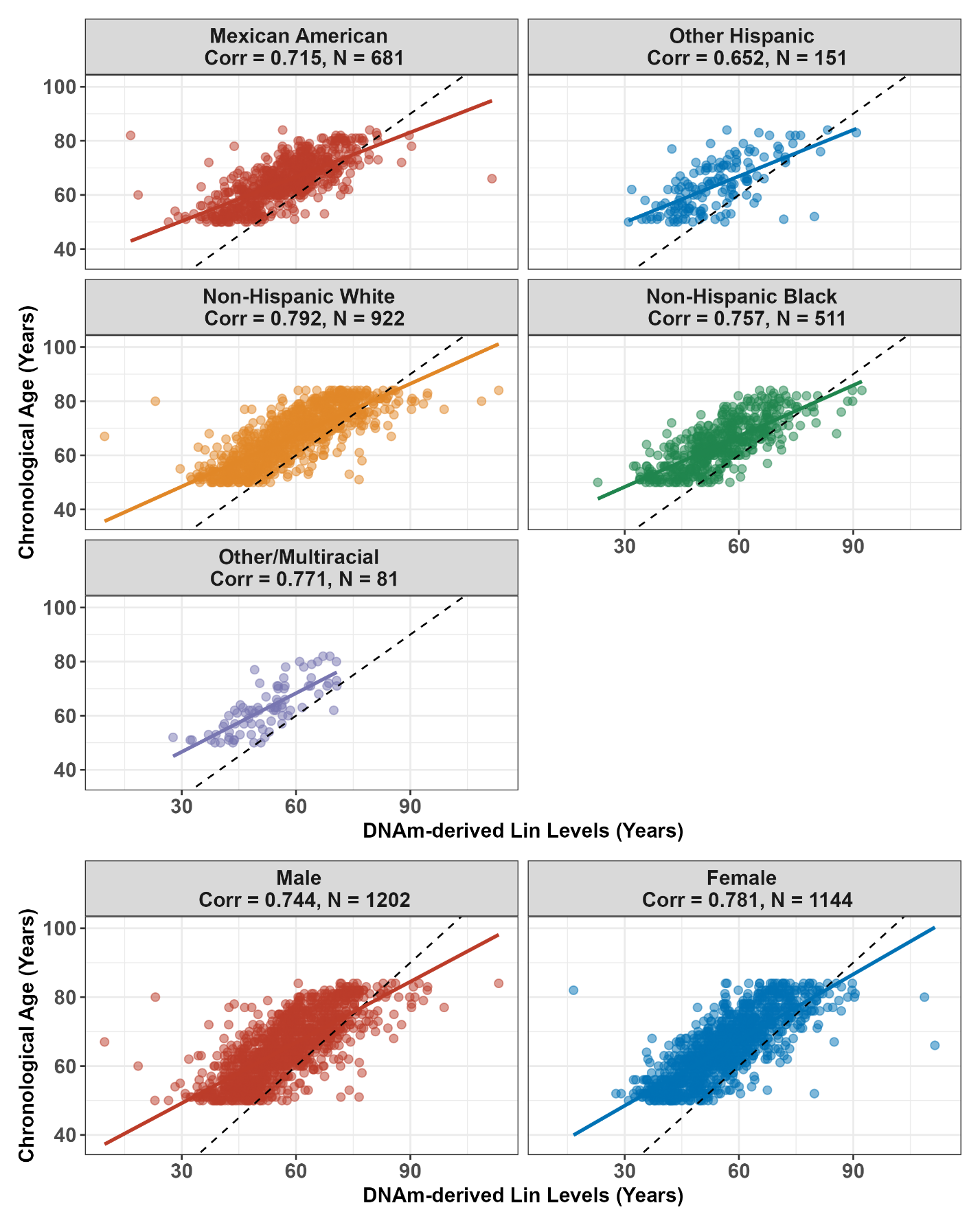
***

***
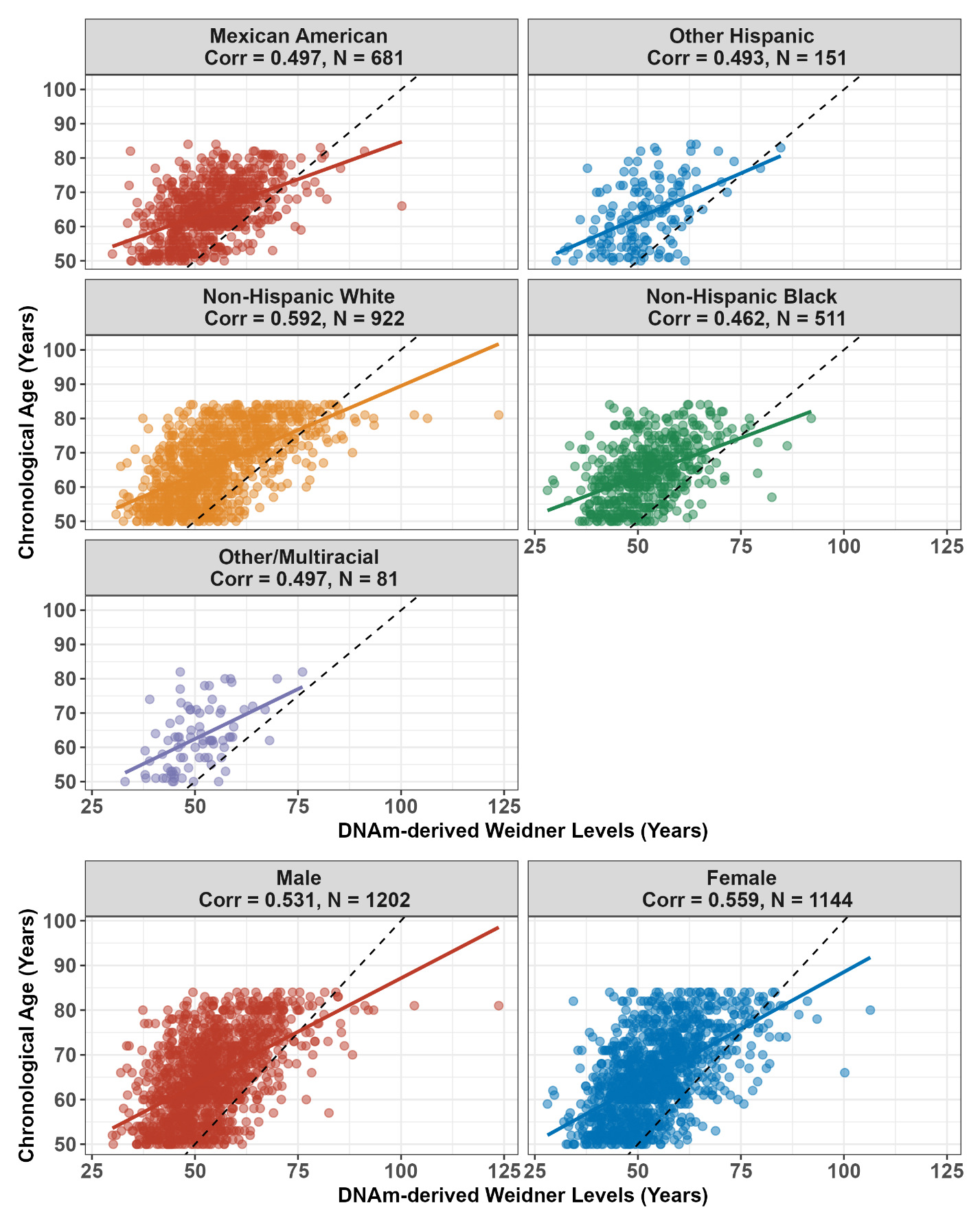
***

***
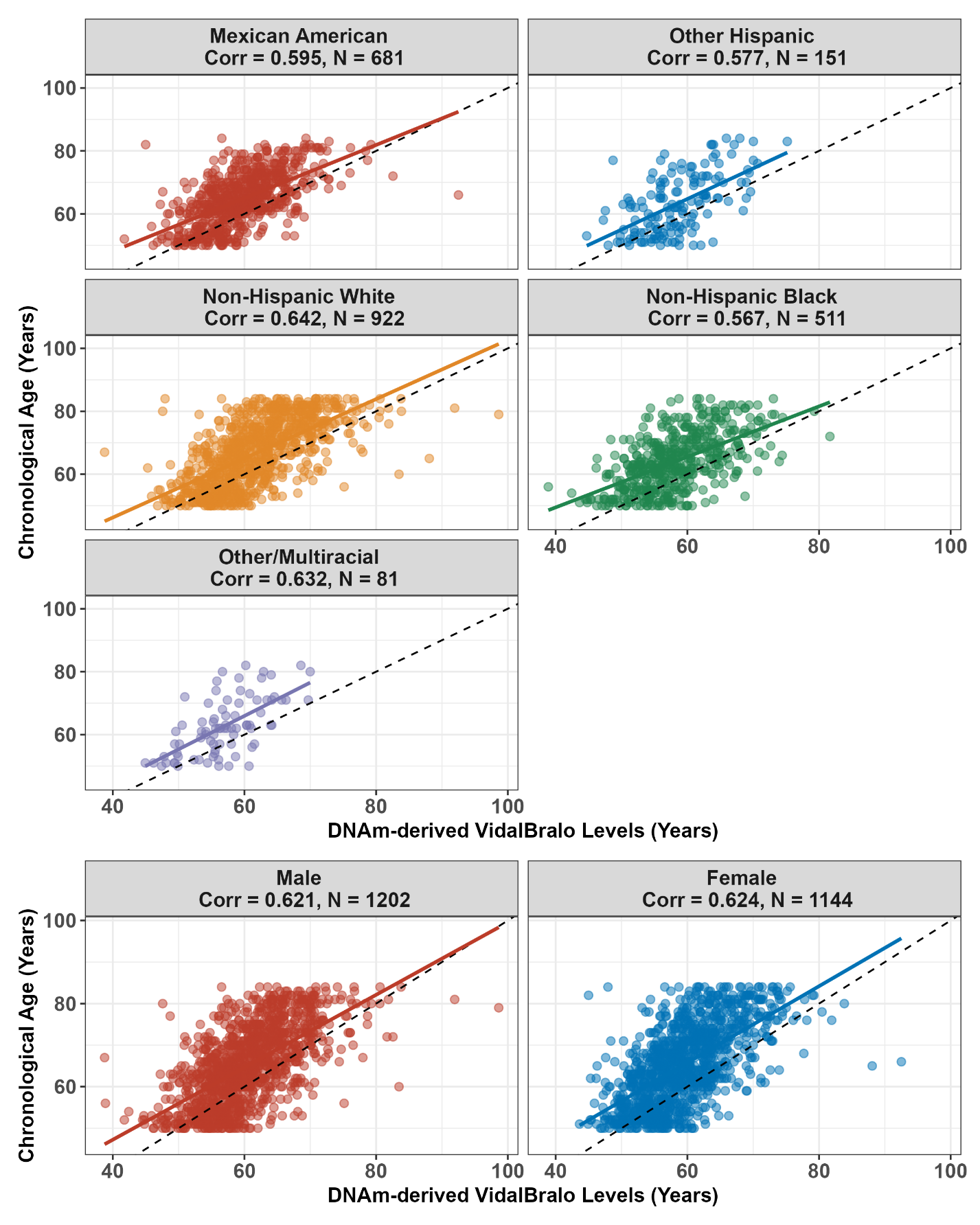
***

***
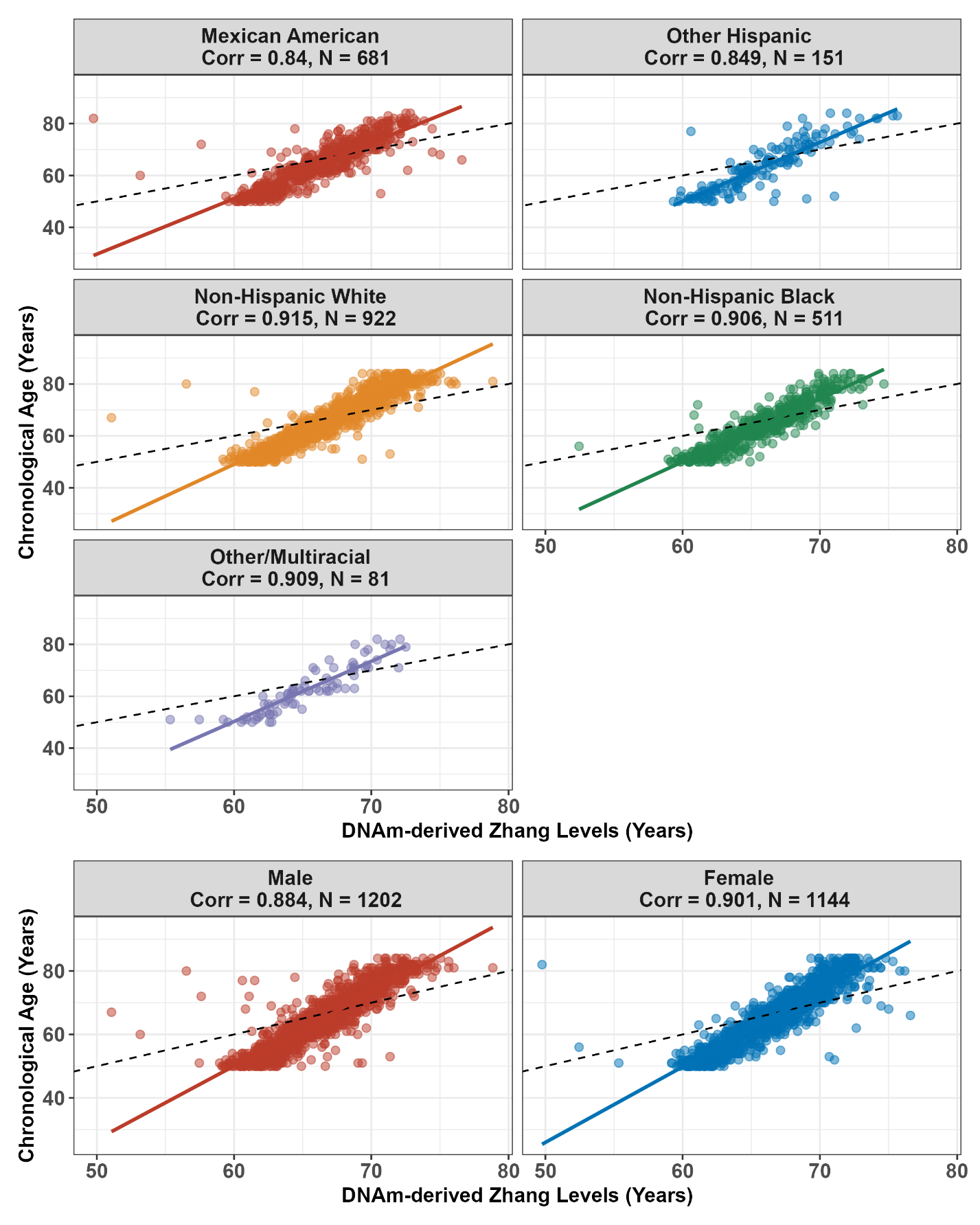
***

***Supplemental Figure 2****: Scatter plots displaying the fit between epigenetic predictions and lab-derived measures or chronological age for each predictor, stratified by race/ethnicity or sex. Dotted line represents the 1:1 fit line, while the colored line corresponds to the best fit linear relationship for the data.*

*
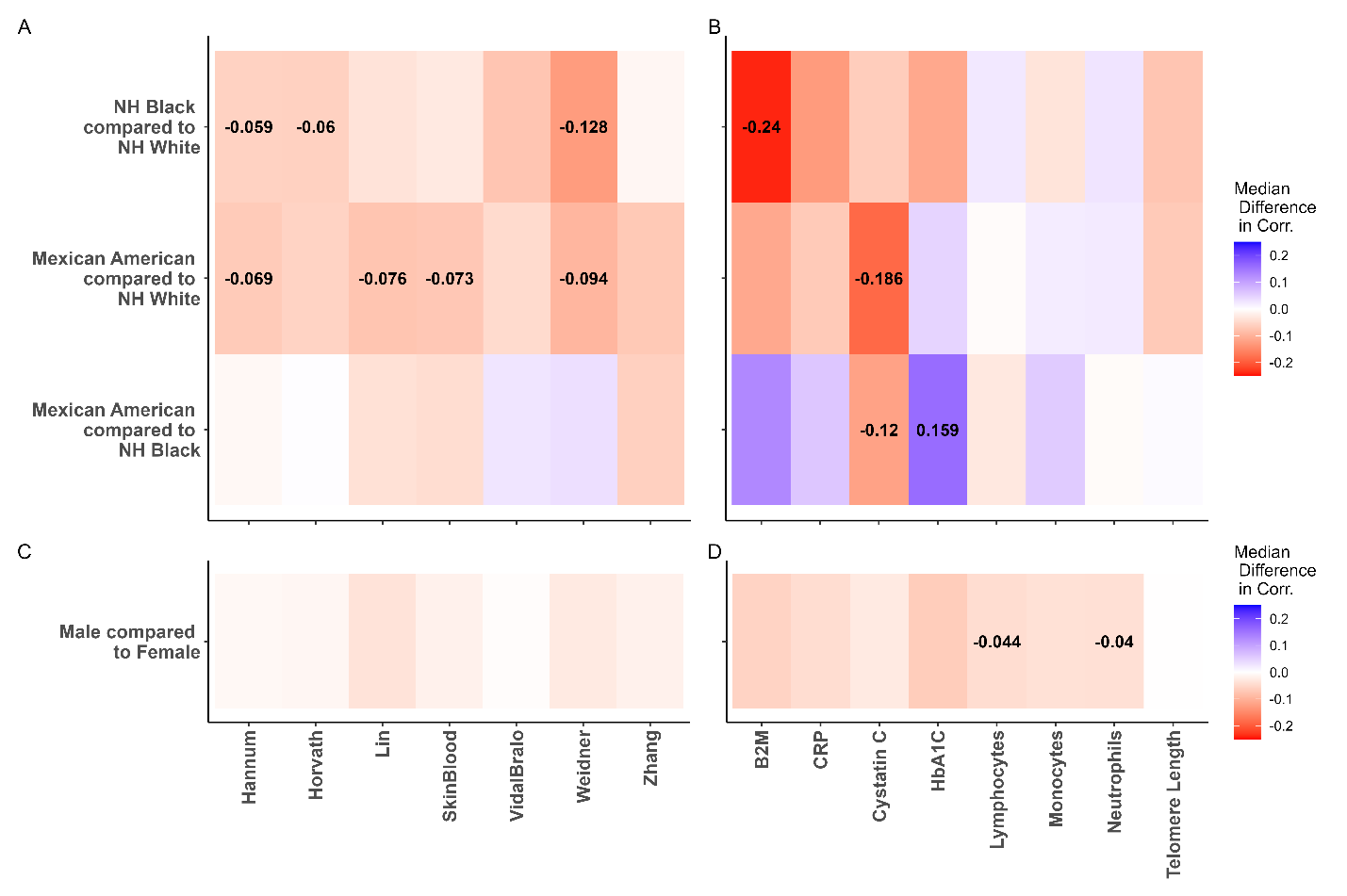
*

***Supplemental Figure 3****:* *Bootstrapped median differences in Pearson Correlation coefficients for epigenetic predictors, stratified by Race/Ethnicity category (A-B) and stratified by sex (C-D). Sensitivity analysis setting all sample sizes equal to the sample size in the smallest group. Color scale denotes median differences in Pearson correlation coefficients from 10,000 iterations of bootstrapping, and median difference in correlation is displayed for significant differences.*


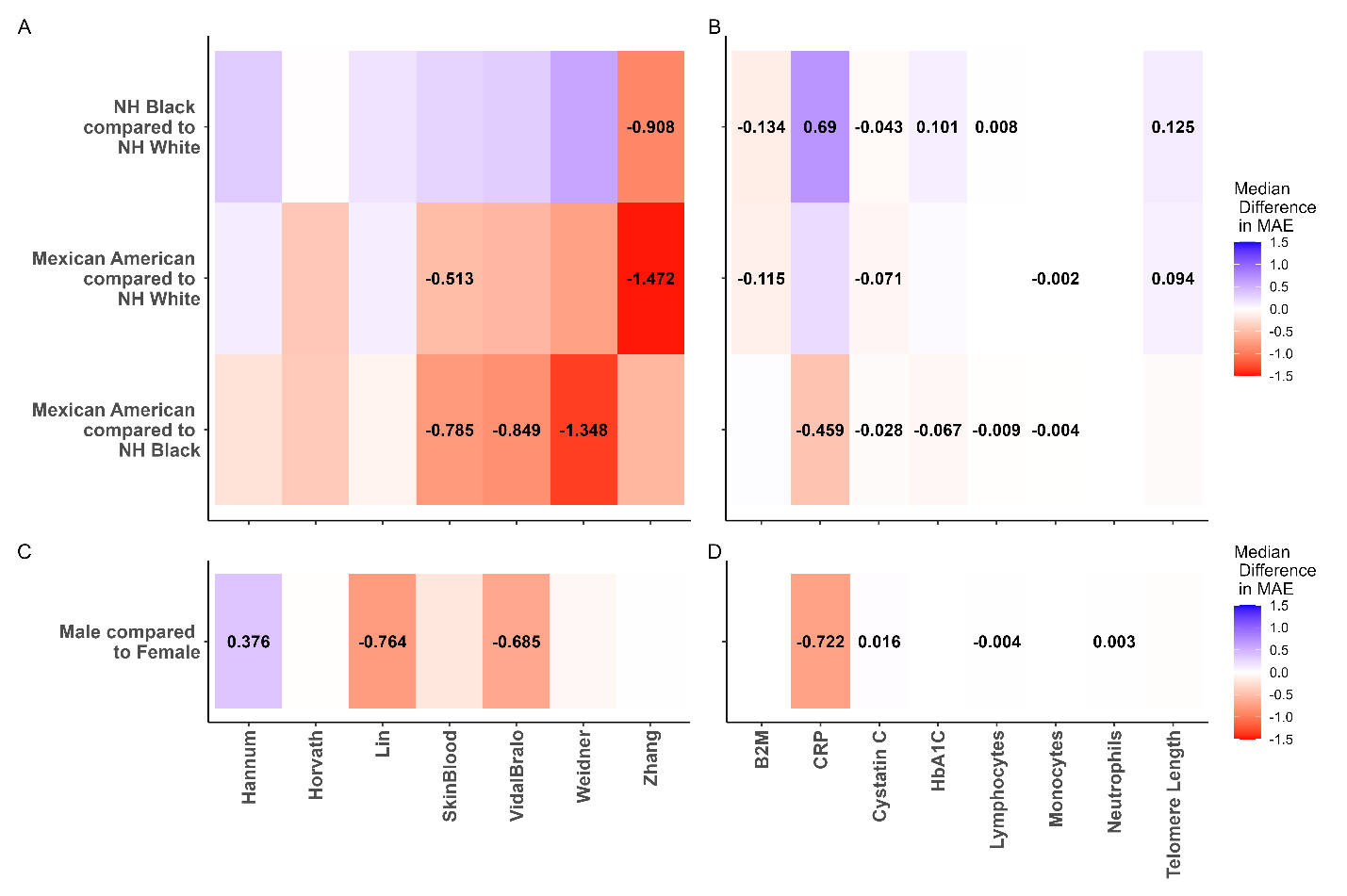


***Supplemental Figure 4****: Bootstrapped median differences in MAE for epigenetic predictors, stratified by Race/Ethnicity category (A-B) and stratified by sex (C-D). Sensitivity analysis setting all sample sizes equal to the sample size in the smallest group. Color scale denotes median differences in MAE from 10,000 iterations of bootstrapping, and median difference in MAE is displayed for significant differences.*

*
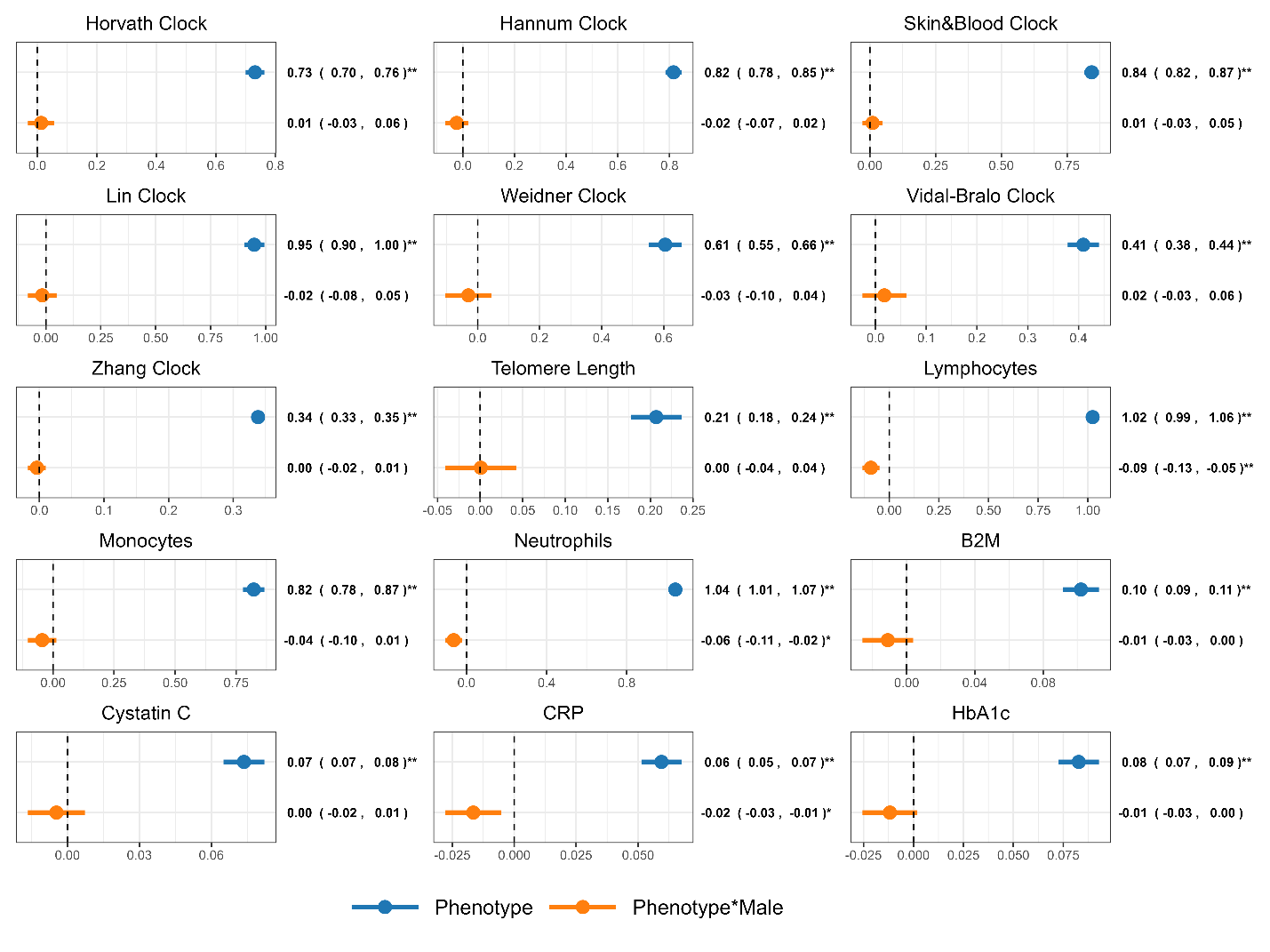
*

***Supplemental Figure 5****: Effect estimates and 95% CIs from multivariate linear regression model summaries (minimally-adjusted) including the epigenetic prediction as the outcome; the phenotypic trait, sex, an interaction term between sex and the phenotypic trait as the predictor variables, further adjusted for race/ethnicity. Phenotype effect estimates (dark blue) reflect the expected change in epigenetic prediction for a 1 unit change in the phenotypic trait within the reference group (female participants). The interaction terms represent the additional expected change in the association between the phenotype and the epigenetic prediction among male participants. “**” indicates a Bonferroni-adjusted p-value < 0.05. “*” indicates an unadjusted p-value < 0.05.*

*
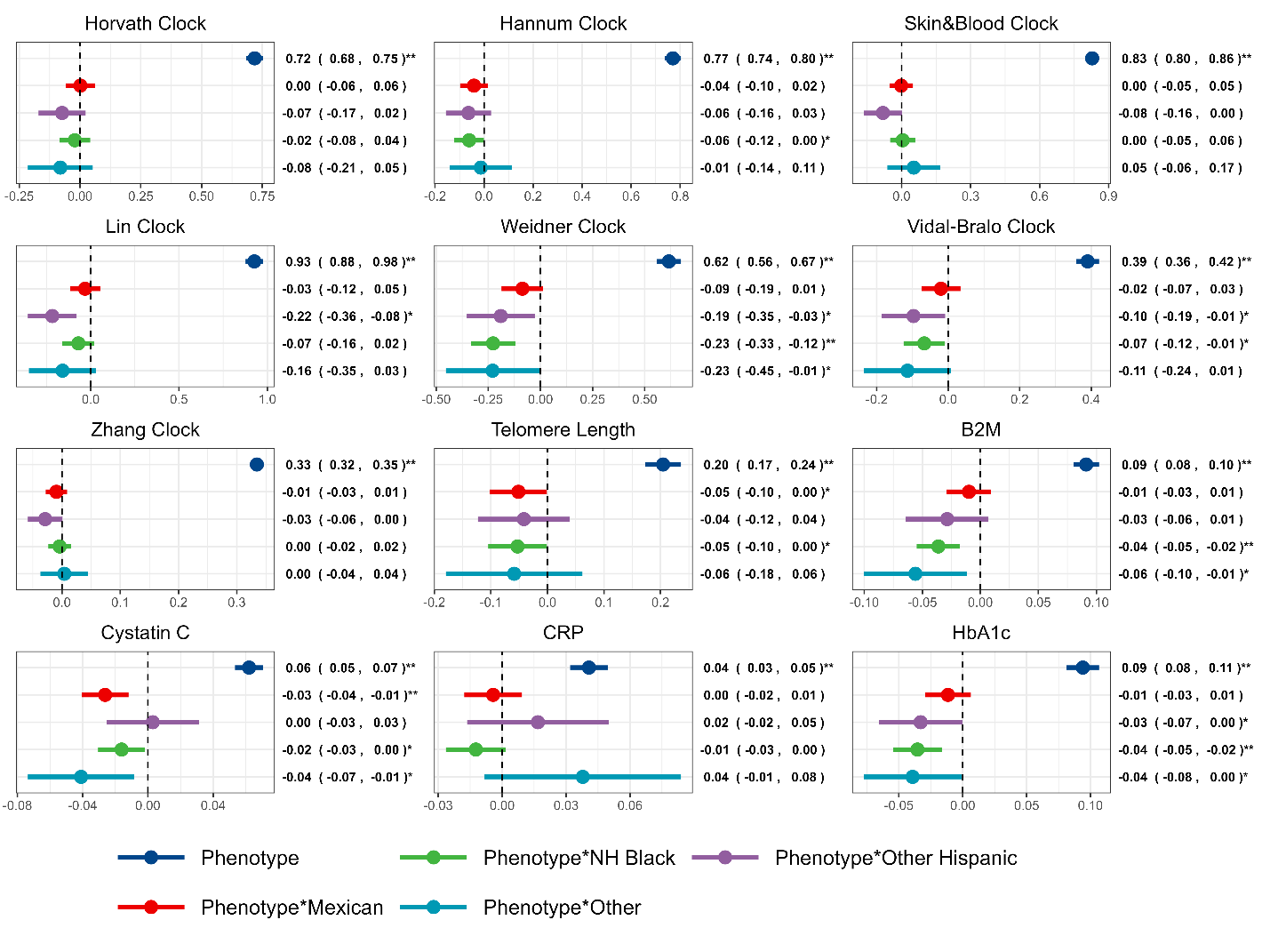
*

***Supplemental Figure 6****: Effect estimates and 95% CIs from multivariate linear regression model summaries (fully-adjusted) including the epigenetic prediction as the outcome; the phenotypic trait, race/ethnicity, an interaction term between race/ethnicity and the phenotypic trait as the predictor variables, further adjusted for sex, education, poverty-to-income ratio, and cell proportions. Phenotype effect estimates (dark blue) reflect the expected change in epigenetic prediction for a 1 unit change in the phenotypic trait within the reference group (NH White participants). The interaction terms represent the additional expected change in the association between the phenotype and the epigenetic prediction within the specified group. “**” indicates a Bonferroni-adjusted p-value < 0.05. “*” indicates an unadjusted p-value < 0.05.*

*
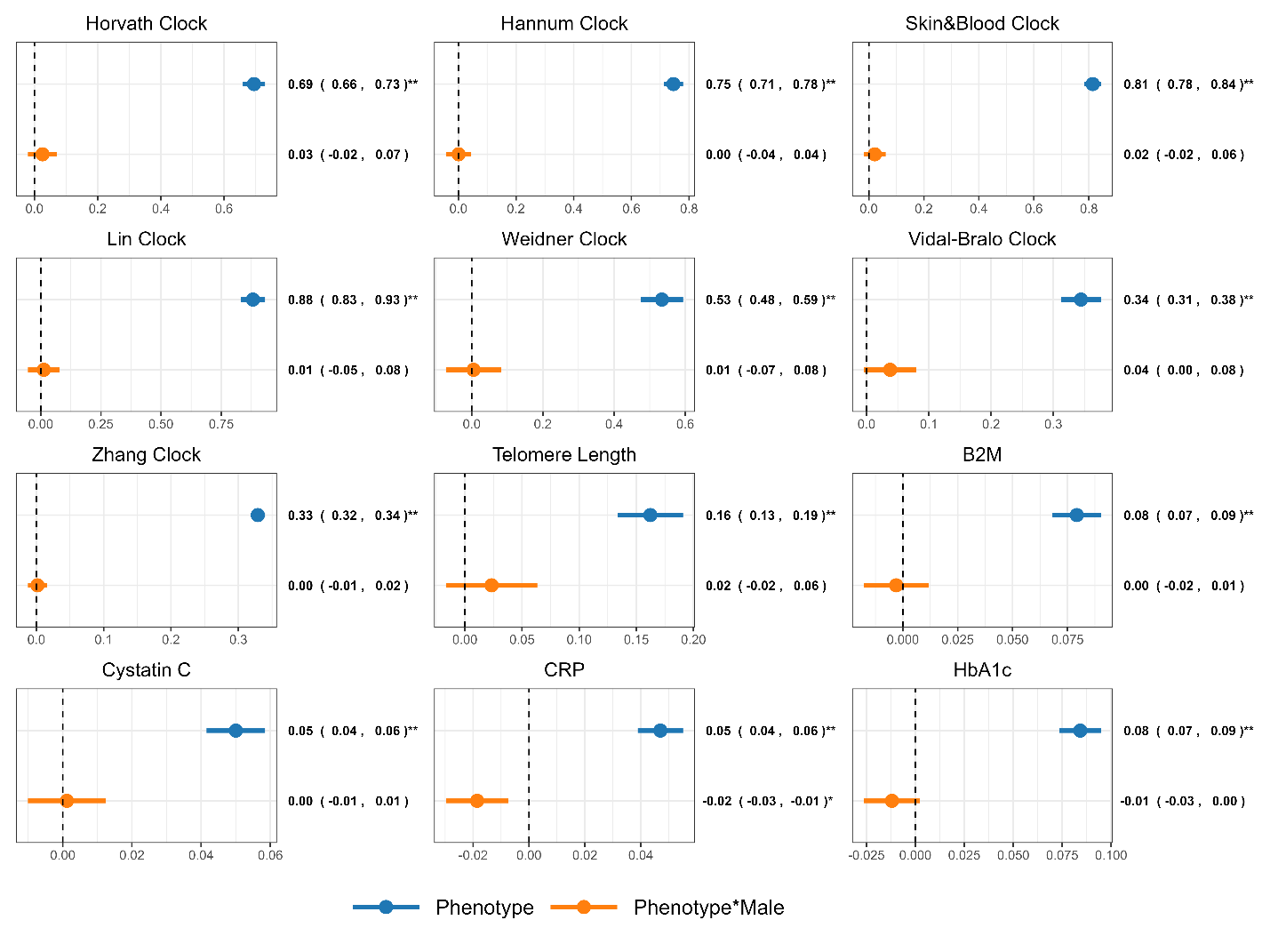
*

***Supplemental Figure 7****: Effect estimates and 95% CIs from multivariate linear regression model summaries (fully-adjusted) including the epigenetic prediction as the outcome; the phenotypic trait, sex, an interaction term between sex and the phenotypic trait as the predictor variables, further adjusted for race/ethnicity, education, poverty-to-income ratio, and cell proportions. Phenotype effect estimates (dark blue) reflect the expected change in epigenetic prediction for a 1 unit change in the phenotypic trait within the reference group (female participants). The interaction terms represent the additional expected change in the association between the phenotype and the epigenetic prediction among male participants. “**” indicates a Bonferroni-adjusted p-value < 0.05. “*” indicates an unadjusted p-value < 0.05.*
